# Projecting the development of antimicrobial resistance in *Neisseria gonorrhoeae* from antimicrobial surveillance data: a mathematical modelling study

**DOI:** 10.1186/s12879-023-08200-4

**Published:** 2023-04-20

**Authors:** Julien Riou, Christian L. Althaus, Hester Allen, Michelle J. Cole, Yonatan H. Grad, Janneke C. M. Heijne, Magnus Unemo, Nicola Low

**Affiliations:** 1 Institute of Social and Preventive Medicine, University of Bern, Bern, Switzerland; 2 UK Health Security Agency, London, UK; 3 Harvard T.H. Chan School of Public Health, Boston, USA; 4 Centre for Infectious Diseases Control, National Institute for Public Health and the Environment, Bilthoven, The Netherlands; 5 Department of Social Medicine, Care and Public Health Research Institute (CAPHRI), Maastricht University, Maastricht, The Netherlands; 6 University of Örebro, Örebro, Sweden

**Keywords:** *Neisseria gonorrhoeae*, Antimicrobial resistance, Minimum inhibitory concentration, Surveillance, Mathematical model

## Abstract

**Background:**

The World Health Organization recommends changing the first-line antimicrobial treatment for gonorrhoea when ≥ 5% of *Neisseria gonorrhoeae* cases fail treatment or are resistant. Susceptibility to ceftriaxone, the last remaining treatment option has been decreasing in many countries. We used antimicrobial resistance surveillance data and developed mathematical models to project the time to reach the 5% threshold for resistance to first-line antimicrobials used for *N. gonorrhoeae*.

**Methods:**

We used data from the Gonococcal Resistance to Antimicrobials Surveillance Programme (GRASP) in England and Wales from 2000–2018 about minimum inhibitory concentrations (MIC) for ciprofloxacin, azithromycin, cefixime and ceftriaxone and antimicrobial treatment in two groups, heterosexual men and women (HMW) and men who have sex with men (MSM). We developed two susceptible-infected-susceptible models to fit these data and produce projections of the proportion of resistance until 2030. The single-step model represents the situation in which a single mutation results in antimicrobial resistance. In the multi-step model, the sequential accumulation of resistance mutations is reflected by changes in the MIC distribution.

**Results:**

The single-step model described resistance to ciprofloxacin well. Both single-step and multi-step models could describe azithromycin and cefixime resistance, with projected resistance levels higher with the multi-step than the single step model. For ceftriaxone, with very few observed cases of full resistance, the multi-step model was needed to describe long-term dynamics of resistance. Extrapolating from the observed upward drift in MIC values, the multi-step model projected ≥ 5% resistance to ceftriaxone could be reached by 2030, based on treatment pressure alone. Ceftriaxone resistance was projected to rise to 13.2% (95% credible interval [CrI]: 0.7–44.8%) among HMW and 19.6% (95%CrI: 2.6–54.4%) among MSM by 2030.

**Conclusions:**

New first-line antimicrobials for gonorrhoea treatment are needed. In the meantime, public health authorities should strengthen surveillance for AMR in *N. gonorrhoeae* and implement strategies for continued antimicrobial stewardship. Our models show the utility of long-term representative surveillance of gonococcal antimicrobial susceptibility data and can be adapted for use in, and for comparison with, other countries.

**Supplementary Information:**

The online version contains supplementary material available at 10.1186/s12879-023-08200-4.

## Introduction

*Neisseria gonorrhoeae* has developed resistance to multiple antibiotic classes, leading to cases of ineffective treatment and the potential for untreatable gonorrhoea [1–3]. The World Health Organization (WHO) estimates that there were more than 82 million new gonorrhoea cases among adults aged 15–49 years in 2020, globally, mostly in low and middle-income countries [4]. Untreated gonorrhoea can cause pelvic inflammatory disease, infertility, and ectopic pregnancy in women, epididymo-orchitis in men, and neonatal ophthalmia [5, 6]. Empirical treatment of people with symptoms suggestive of gonorrhoea, regardless of knowledge of the aetiological agent or antimicrobial resistance (AMR) profile, is encouraged to limit the further spread of *N. gonorrhoeae* and occurrence of complications. WHO suggests that the first-line regimen for empirical treatment in a given country should have a microbiological cure rate > 95% and should be changed when ≥ 5% of cases fail treatment or ≥ 5% of tested gonococcal isolates are resistant [7]. In many countries, AMR levels for previously and currently recommended first-line antimicrobials exceed 5% [8].

The injectable extended spectrum cephalosporin (ESC), ceftriaxone, is recommended for empirical treatment of suspected gonorrhoea in most countries [9]. The development of resistance to ceftriaxone and other extended-spectrum cephalosporins (ESC) is a complex process, most frequently involving multiple mechanisms (‘multi-step’ resistance) [10–12]. Sporadic cases of treatment failure due to ceftriaxone resistance have been reported since 2009 from Japan, Australia, Sweden, Slovenia, Austria and the UK [13–15]. However, surveillance systems, such as the Gonococcal Resistance to Antimicrobials Surveillance Programme (GRASP) in England and Wales, show that isolates above the cut-off for resistance to ceftriaxone remain rare and are may have been imported in association with travel [16]. The distribution of ceftriaxone minimum inhibitory concentrations (MICs) drifted upwards until 2018, but might have stabilised or reversed since 2019 [10, 16]. The overall number of reported gonorrhoea cases and number of GRASP isolates declined in 2020, during the coronavirus disease 2019 (COVID-19) pandemic [16]. MIC drift can be interpreted as a progressive accumulation of mutations in response to the selection pressure exerted by the continued use of ESCs, including the suboptimal use of oral ESCs, such as cefixime, which is expressed phenotypically as an increase in MICs [17]. There is no licensed antimicrobial available yet that could replace ceftriaxone once AMR levels exceed the 5% threshold.

Projections about the probable time when the 5% threshold for resistance might be reached could help public health agencies to improve strategies for dealing with AMR in *N. gonorrhoeae.* Mathematical modelling studies provide insights into the emergence and spread of AMR and have been used to model the emergence of and future spread of resistance in *N. gonorrhoeae* [17–19] and *Mycoplasma genitalium,* and have also shown that higher levels of antibiotic treatment for *N. gonorrhoeae* result in faster development of AMR [17, 20]. The modelling studies of *N. gonorrhoeae* focused on resistance to ciprofloxacin, in which the presence of a single point mutation in the *gyrA* gene (GyrA 91F, often seen together with GyrA 95) can result in clinical resistance and treatment failure, so-called single-step resistance (2). The consideration of the development of AMR as a binary property [17–19] may be an appropriate simplification for the study of resistance to antimicrobials like ciprofloxacin, but may be inadequate for antimicrobial classes such as ESCs with more complicated patterns of AMR development [10, 17, 21]. AMR surveillance systems that report the MIC for each tested isolate, and the antibiotic dispensed, provide more information about decreases in susceptibility than binary data about isolates with full resistance. The overall aim of this study was to model the development of AMR in *N. gonorrhoeae* by linking data from a national surveillance system that records MIC values and antibiotic treatment. The main outputs are projections for when the proportion of *N. gonorrhoeae* that is resistant to ceftriaxone will reach the 5% threshold, should current trends continue.

## Methods

### Surveillance data

GRASP is a sentinel surveillance system in England and Wales, established in 2000 and administered by the UK Health Security Agency (formerly Public Health England). Between July and September every year, *N. gonorrhoeae* isolates, together with clinical and behavioural information including gender, sexual orientation and antibiotic treatment, are collected from consecutive individuals attending a network of collaborating sexual health clinics [22]. The laboratories associated with the clinics test isolates for susceptibility to eight different antibiotics, including ciprofloxacin, azithromycin and the ESCs cefixime and ceftriaxone [22].

The GRASP data were aggregated by year and by the number of *N. gonorrhoeae* isolates in each MIC doubling dilution class for ciprofloxacin (data available for the years 2000–2018), azithromycin (2001–2018), cefixime (2004–2018) and ceftriaxone (2004–2018), stratified by presumed sexual orientation (heterosexual men and women, HMW) or behaviour (men who have sex with men, MSM) (Additional file 1). These four antibiotics were chosen as they have all been part of the UK national recommendations since GRASP began (ciprofloxacin until 2005, cefixime 2005–2010, and ceftriaxone in combination with azithromycin from 2010–2018). We also used antibiotic treatment data from GRASP to derive proportions of patients with a diagnosis of gonorrhoea who were prescribed each antibiotic, aggregated by year, gender and presumed sexual orientation. We defined resistance using European Committee on Antimicrobial Susceptibility Testing (EUCAST) clinical resistance breakpoints version 9.0 for ciprofloxacin (MIC > 0.6 mg/L), cefixime (MIC > 0.125 mg/L) and ceftriaxone (MIC > 0.125 mg/L), and the epidemiological cut-off value (MIC > 1 mg/L) for azithromycin [23]. Discrete prescription data by year for HMW or MSM were smoothed by time-continuous functions *p(t)* to be used in an ordinary differential equation framework, which is continuous (Additional file 2).

### Modelling resistance acquisition

The central idea of the models in this study is to link the development of resistance to the level of antibiotic use in a mechanistic susceptible-infected-susceptible (SIS) framework. We report our approach starting with a model in which AMR is described by a single-step process.

Resistance as a single-step process.

**Fig. 1:**
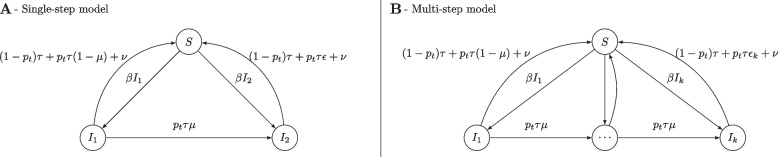
Schematic descriptions of the models for the development of antimicrobial resistance in Neisseria gonorrhoeae. Panel **A**, single-step, panel **B**, multiple steps to resistance. Compartment *S* corresponds to susceptible; compartments *I*_1_ to *I*_k_ correspond to infected with increasingly resistant strains of N. gonorrhoeae. *β* is the transmission rate, *ν* is the rate of spontaneous recovery, *τ* is the rate of recovery due to treatment, *μ* is the probability of developing resistance (or one step of resistance for the multi-step model) upon treatment, and *ϵ* is the reduction of treatment efficacy for resistant strains. The function* p(t)* corresponds to the probability that treatment at time *t* includes the antibiotic of interest

In the single-step model, AMR is a binary characteristic of a bacterium for a given antibiotic (Fig. 1).

The model has two infected compartments: infection by non-resistant bacteria (compartment *I*_*1*_) or by bacteria resistant to the antibiotic of interest (compartment *I*_*2*_). Susceptible individuals (compartment *S*) become infected with either type of bacterium *k* ∈ {1,2}; according to the force of infection *βI*_*k*_. The model describes the population-level dynamics of resistance development in relation to antibiotic usage with five parameters: *{β, ν, τ, μ, ϵ}.* Infected individuals recover spontaneously at rate *ν*, or through treatment at rate *τ*. We introduce treatment data using the forcing function *p(t)*, representing the probability that treatment at time *t* includes the antibiotic of interest. If the antibiotic of interest is prescribed (probability *p(t)*) the effect of treatment differs according to the resistance status. Infections by wild-type bacteria (compartment *I*_*1*_) can either recover through treatment at rate *τ* (with probability 1*- μ*) or develop resistance in one single-step (with probability *μ*). For infections by resistant bacteria (compartment *I*_*2*_), recovery through treatment occurs with a lower efficacy ϵ ∈ [0,1]. If another antibiotic is prescribed (probability 1—*p(t)*), recovery through treatment occurs at the same rate *τ* for compartments* I*_*1*_ and *I*_*2*_. We assume no fitness cost for resistance here, as we focus on the dynamics of resistance under treatment pressure. Within the chosen framework, a fitness cost would only scale with the mutation probability under treatment pressure, and lead to a decrease of resistance when treatment pressure is removed.

### Resistance as a multi-step process

We developed an alternative, multi-step model, using the same five parameters: *{β, ν, τ, μ, ϵ}* (Fig. 1). This model considers the acquisition of resistance as a progressive process in which resistance mutations accumulate. The model is particularly appropriate for describing the dynamics of AMR to antimicrobials for which a drift in MIC is observed, but for which very few isolates have an MIC value above the EUCAST cut-off for resistance, such as ceftriaxone. The model structure is similar to the single-step model but includes multiple infected compartments *{I*_*1*_*, …, I*_*k*_*}*. These *k* compartments correspond to the *k* MIC classes reported in GRASP (8 in the case of ceftriaxone, 9 for cefixime and 12 for azithromycin) and represent increasing levels of AMR. This model has two central assumptions. First, the probability of developing an additional step of resistance upon treatment *μ* is the same for every class, in the absence of empirical evidence to the contrary. Second, increasing levels of AMR lead to a linear decrease in treatment efficacy from *ϵ*_*1*_ = 1 to *ϵ*_*k*_ ∈ [0; 1] (estimated).

### Implementation and inference

We implemented both models in Stan, an open-source general purpose inference software for a large range of Bayesian models, and ran the models in R using package rstan [24]. We used four MCMC chains with 2,000 iterations each, and burned in the first 1,000, for a total of 4,000 posterior samples. We assumed that *N. gonorrhoeae* prevalence was stable (i.e., at endemic equilibrium) in 2000, with values ranging between 0.16% and 0.38% in HMW and between 1.19% and 2.79% in MSM [17]. In a sensitivity analysis, we allowed the prevalence to increase over time according to different scenarios. We simultaneously modelled the MSM and HMW populations with common parameters for spontaneous recovery *ν*, resistance mutation probability *μ*, and treatment efficacy* ϵ*. Other parameters such as initial prevalence, initial resistance and treatment rate were considered independently for each population in the joint model. The models were fitted with a Markov chain Monte Carlo method using beta-binomial (single-step model) or dirichlet-multinomial (multi-step model) likelihoods, accounting for over-dispersion. The single-step model was fitted to GRASP data about the proportion of resistant isolates and the multi-step model was fitted to the data about the proportions of isolates at each MIC doubling dilution. We applied both models separately to data over the period 2000–2018. For ciprofloxacin, we only used the single-step model because a) the mechanism of ciprofloxacin resistance acquisition with single point mutations is best described by the single-step model and b) laboratory reporting of MIC data changed after 2009 (Additional file 1). For ceftriaxone, cefixime and azithromycin, we applied both single-step and multi-step models. We used the fitted models to project the dynamics of resistance in response to assumptions of antibiotic usage from 2000 to 2030. In sensitivity analyses, we removed the assumption of endemic equilibrium in 2000, and considered different scenarios of increasing prevalence. More details about the model are available in Additional file 3. For each parameter, we report the estimate of the median of the posterior distribution together with the 2.5% and 97.5% quantiles (forming the 95% credibility interval [95%CrI]) computed from the posterior samples. More details about the model fit are shown in Additional file 4, and all parameter estimates are shown in Additional file 5. Code is available from https://github.com/jriou/ngmic.

## Results

The GRASP data from 2000–2018 included results for 15,699 individual *N. gonorrhoeae* isolates from HMW and 12,368 isolates from MSM. Of these, we retained a total of 26,437 isolates with information on MIC for ciprofloxacin, 25,626 for azithromycin, 19,403 for cefixime and 19,424 for ceftriaxone. Additional file 1 shows detailed data about proportions of resistant isolates and MIC distributions and additional file 2 shows the use of each antibiotic.

### Ciprofloxacin

**Fig. 2:**
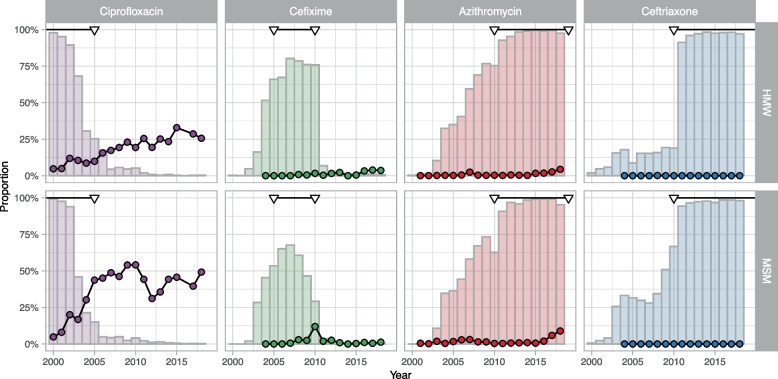
Antibiotic prescriptions for Neisseria gonorrhoeae infection and proportion of resistance to different antibiotics. Prescriptions and resistance are shown with bars and circles, respectively, in heterosexual men and women (HMW) and men who have sex with men (MSM) from the Gonococcal Resistance to Antimicrobials Surveillance Programme (GRASP) (18) from 2000–2018. Triangles indicate the period during which each antibiotic was recommended as a first-line empirical treatment

**Fig. 3:**
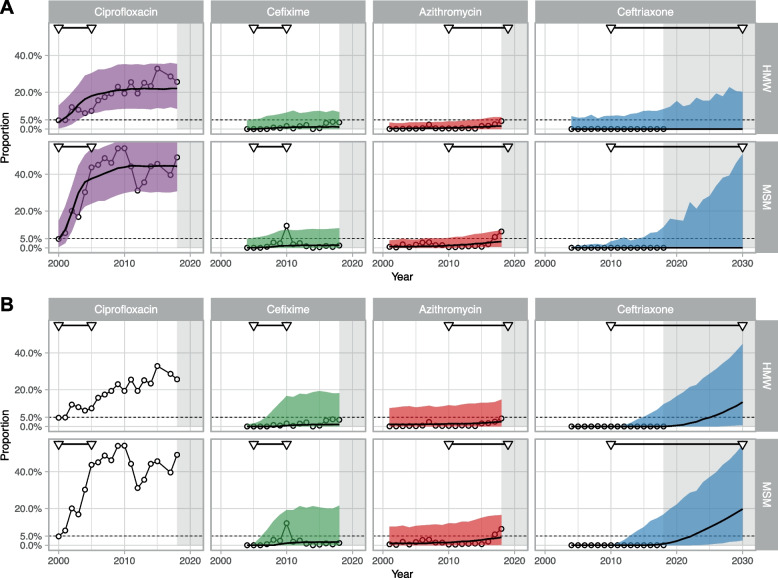
Projected levels of antibiotic resistance from 2000 to 2018 (for ciprofloxacin, cefixime and azithromycin) or 2030 (for ceftriaxone). Panel **A**, single-step model, panel **B**, multi-step model in heterosexual men and women (HMW) and men who have sex with men (MSM). The lines correspond to median projections and the shaded areas to 95% prediction intervals. The 5% threshold is shown by the dotted line. Circles show the proportion of resistant isolates in the GRASP data. Triangles indicate the period during which each antibiotic has been recommended or is predicted to be recommended (ceftriaxone) as a first-line empirical treatment

Ciprofloxacin was used to treat most people included in GRASP in 2000 and usage decreased from around 2004 to almost zero by 2018 (Fig. 2). The proportions of GRASP isolates with ciprofloxacin resistance (MIC > 0.06 mg/L) increased from 2000 onwards. As ciprofloxacin use decreased during the second half of the 2010s, the proportion of resistant isolates stabilised in MSM, but continued to increase in HMW until 2015. The single-step model described the general trend of ciprofloxacin AMR from 2000–2018 well. Using projections from the single-step model, which links observed proportions of resistance to antibiotic use, we estimate that, between 2000 and 2018, resistance to ciprofloxacin rose from 3.7% (95%CrI: 0.3–12.7%) to 22.0% (95%CrI: 11.0–35.4%) in HMW and from 4.6% (95% CrI: 0.4–14.6%) to 44.4% (95% CrI: 30.8–59.6%) in MSM (Fig. 3A, detailed results in Additional file 4). The observed proportions of resistant samples in GRASP in 2018 were 26% (142/554, 95% CI: 22–29%) in HMW and 49% (393/799, 95% CI: 46–53%) in MSM.

### Azithromycin

GRASP data report an increase in azithromycin use from 2003 to 2010. After its inclusion, in combination with ceftriaxone, in the national recommendations, it was used in almost all cases from 2010 to 2018 (Fig. 2). It was removed from the recommended regimen in 2018. Between 2001 and 2018, we observed an increase in the proportion of isolates with azithromycin resistance (MIC > 1 mg/L). Using the single-step model, we estimated an increase from 0.3% (95% CrI: 0.0–3.6%) to 1.6% (95% CrI: 0.0–6.5%) in HMW and from 0.7% (95% CrI: 0.0–4.0%) to 3.4% (95% CrI: 0.5–9.5%) in MSM (Fig. 3A, detailed results in Additional file 4).

The proportions of GRASP isolates at each MIC doubling of azithromycin appear relatively stable over time, with a sudden increase around 2015 (Additional file 1). Estimates of resistance from the multi-step model, making use of these MIC data, predict slightly higher levels of resistance than with the single-step model, at 2.5% (95% CrI: 0.0–14.6%) for HMW and 4.3% (95% CrI: 0.3–16.4%) for MSM in 2018 (Fig. 3B, Additional file 4). The observed proportions of resistant samples in GRASP data in 2018 were 4% (24/554, 95% CI: 3–6%) in HMW and 9% (71/799, 95% CI: 7–11%) in MSM.

### Cefixime

Cefixime use increased from around 2003–2007, replacing ciprofloxacin (Fig. 2). About 75% of HMW included in GRASP were treated with cefixime from 2007–2010. The proportion of MSM receiving cefixime started to decrease from 2007. GRASP data show a small increase in the proportion of isolates with cefixime resistance (MIC > 0.125 mg/L) in HMW up to 2010. In MSM, the proportion of cefixime resistance increased abruptly to 12% (65/543) for one year in 2010, then decreased again, coincident with the introduction of the dual therapy regimen. The MIC distributions for cefixime show a slight shift for HMW and MSM (Additional file 1). This general trend is perturbed by a large downward shift of MIC levels in 2006, and by a temporary upward shift of MIC levels around 2009–2010, mostly among MSM. With the single-step model, we estimate that between 2004 and 2010, cefixime resistance would increase from 0.0% (95% CrI: 0.0–4.6%) to 0.8% (95% CrI: 0.0–8.9%) in HMW and from 0.0% (95% CrI: 0.0–4.8%) to 1.1% (95% CrI: 0.0–9.8%) in MSM, then stabilise around these values (Fig. 3A, detailed results in Additional file 4). The model did not capture the outlier of 2010. Applying the multi-step model to MIC data on cefixime the model leads to very similar projections for the period 2004–2018, although with increased uncertainty (Fig. 3B, detailed results in Additional file 4).

### Ceftriaxone

**Fig. 4:**
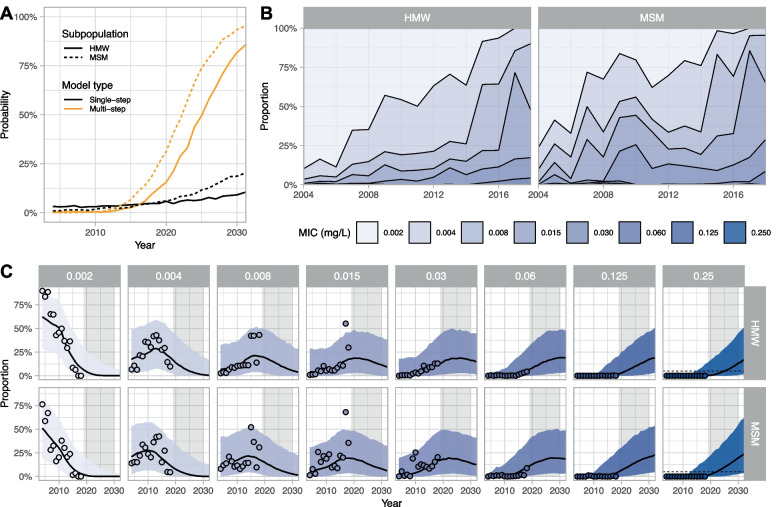
Probability of ceftriaxone resistance and observed and modelled MIC drift. (A) Projected probability that ceftriaxone resistance will reach 5% over the period 2000–2030 in heterosexual men and women (HMW) and men who have sex with men (MSM), according to the single-step or the multi-step model. (B) MIC data for ceftriaxone from the Gonococcal Resistance to Antimicrobials Surveillance Programme (GRASP) (18) over 2000–2018. (C) Fit of the multi-step model to MIC data for ceftriaxone. The lines correspond to median projection and the shaded areas to 95% prediction intervals. Circles correspond to GRASP MIC data

Ceftriaxone use increased from around 2003 and was used at a low level (slightly higher among MSM than among HMW) until 2010. In 2010, it became part of the recommended first-line dual drug regimen and has been used in almost all cases included in GRASP since then (Fig. 2). The GRASP dataset includes only one case of resistance (MIC > 0.125 mg/L), in 2008. Accordingly, the single-step model estimates a low stable proportion of resistance in 2018, at 0.0% (95% CrI: 0.0–10.8%) for HMW and 0.0% (95% CrI: 0.0–11.3%) for MSM. Assuming a continuation of universal use of ceftriaxone, the single-step model projects very low levels of resistance in 2030, at 0.0% (95% CrI: 0.0–20.2%) for HMW and 0.0% (95%CrI: 0.0–51.0%) for MSM, but with considerable uncertainty. This translates into a probability of reaching the 5% threshold by 2030 of 9.1% for HMW and 18.6% for MSM (Fig. 4A).

The GRASP data show a clear drift in MIC values for ceftriaxone in both HMW and MSM from 2005 (Fig. 4B). As for cefixime, we observed a temporary upward shift of MIC levels around 2009–2010 among MSM. The multi-step model captures this MIC drift (Fig. 4C), resulting in slightly higher estimates for the proportion of resistance in 2018, at 0.0% (95% CrI: 0.0–12.1%) for HMW and 0.8% (95% CrI: 0.0–16.8%) for MSM. Projections for 2030 are also higher than in the single-step model, at 13.2% (95% CrI: 0.7–44.8%) for HMW and 19.6% (95%CrI: 2.6–54.4%) for MSM. These projected values translate into high probabilities of reaching the 5% threshold by 2030 of 82% for HMW and 93% for MSM (Fig. 4A). In a sensitivity analysis, ignoring data from the temporary upward shift in 2009–2010, we find that the probability of reaching the 5% threshold by 2030 increases to 92% for HMW and 97% for MSM. Relaxing the assumption of endemic equilibrium, with different scenarios of increase in transmissibility and prevalence, did not impact these results (Additional file 4).

## Discussion

In this study, we developed a mechanistic model to analyse and extrapolate detailed *N. gonorrhoeae* surveillance data about MIC drift and antibiotic treatment, linking the accumulation of resistance mutations to the selective pressure exerted by antibiotics. The multi-step model captured the progressive shift in the MIC values for ceftriaxone well. The drift in MIC values towards decreasing ceftriaxone susceptibility is a longstanding global concern [7, 10, 25]. The GRASP data included only one ceftriaxone-resistant isolate by 2018, so in the single-step model, which relies on the proportions, the projected level did not exceed the 5% AMR threshold at all. Extrapolating from the trend in MICs in the multi-step model predicts that ceftriaxone resistance will likely reach a proportion of 5% in MSM by 2030, if current trends continue, but with a wide credibility interval.

To our knowledge, this is the first mathematical modelling study that describes the sequential accumulation of resistance mutations and integrates data about MIC drift. The Bayesian approach allowed us to propagate the considerable uncertainty in model parameters and to provide meaningful projections of the expected growth in antibiotic resistant *N. gonorrhoeae*. There are limitations to the study. First, the approach assumes that *N. gonorrhoeae* resistance is solely driven by selection pressure from antibiotic usage within the country [17]. Whether de novo acquisition of resistance mutations due to treatment pressure is the main driver of MIC drift remains unknown. *N. gonorrhoeae* can acquire resistance mutations through horizontal gene transfer from reservoirs of AMR genes in commensal *Neisseria* spp. in the pharynx [1–3]. The resulting mosaic *penA* allele, and other mutations e.g. in *mtrR* and *porB1b* confer intermediate resistance to *N. gonorrhoeae* and can together lead to higher level resistance [1, 2, 12]. There is some evidence from studies of *Streptococcus pneumoniae,* demonstrating the evolution of MIC drift to full resistance [26]. Second, the model does not incorporate the importation of resistant strains of *N. gonorrhoeae* from regions with higher frequencies of AMR, such as south east Asia [2, 27]*.* It can be argued, however, that intermittent import of resistant *N. gonorrhoeae* strains that result in treatment failure and spread rapidly would produce a sudden, rather than the observed gradual shifts in MIC, which would then decrease due to enhanced gonorrhoea control measures, clonal replacement or fitness costs. Increasing then decreasing ESC resistance has occurred, including the circulation of a multi-drug resistant clone, NG-MAST ST1407, originating in Japan in 2004 and subsequently spreading widely internationally [27–31]. Introduction of new strains might thus add to the underlying, progressive accumulation of resistance mutations due to domestic treatment pressure. The temporary upward shift in MIC observed around 2009 and 2010 in MSM in the GRASP data supports this interpretation. In the sensitivity analysis in our model, removing observations from 2009 and 2010 led to an even earlier projection of increasing resistance in MSM because the 2009–2010 peak lowered the estimated slope of resistance. Further model developments could account for international spread, but the lack of representative data about AMR in many other countries makes this complicated. Third, our analysis includes GRASP data up to 2018 and the study was delayed by the COVID-19 pandemic. GRASP data collection continued and in 2020 there were no ceftriaxone-resistant samples and MIC values had decreased slightly [16]. The representativeness of the GRASP data might also have been affected by the pandemic conditions, given that the number of isolates and total number of diagnosed cases declined. Including data up to 2018 is therefore reasonable but, if a downward trend in MICs continues, the projected probability of AMR > 5% in the multi-step model would be reduced. Fourth, there are more GRASP collaborating centres in London than in other regions. If levels of gonorrhoea and AMR are higher in London, extrapolation of the rate of projected spread of AMR might be overestimated at the national level.

Our results rely upon several additional assumptions. First, increasing levels of AMR are assumed to lead to a linear decrease in treatment efficacy, although some non-linear effects are likely. A more abrupt decrease in treatment efficacy at the resistance threshold would lead to higher future levels of resistance. Second, we did not incorporate the effects of fitness costs and epistasis, which might be especially important when considering long-term resistance to antibiotics that are no longer in use such as ciprofloxacin. For this reason, we only propose long-term forecasts for ceftriaxone, assuming continuous use, as it is the net effect of treatment pressure and fitness costs that matters in this case. Third, we only considered antibiotics used to treat gonorrhoea; antibiotics may exert selective pressure when prescribed for other infections, which could partly explain persistent high levels of ciprofloxacin resistance [32]. Finally, we used deterministic models that ignore the role of stochastic events but accounted for the additional uncertainty by using over-dispersed probability distributions (beta-binomial and dirichlet-multinomial).

Our study and its findings have implications for future strategies for gonococcal prevention, treatment and surveillance. Our projections suggest a window of ten or more years before resistance to ceftriaxone exceeds 5% in England and Wales, which provides some time for planning strategies for continued antimicrobial stewardship and developing and evaluating new antimicrobials. New diagnostic tests might allow resistance-guided therapy for the 50–70% of isolates that are still susceptible to ciprofloxacin [33]. Phase 3 randomised clinical trials are evaluating newly developed antibiotics, including zoliflodacin, and existing antibiotics [34, 35]. Development of a gonococcal vaccine as primary prevention could delay the emergence of AMR if uptake is high enough, although the technical development pathway is even less advanced than that for new antimicrobials [36]. Our models could also be extended to explore the potential impact of new diagnostic and treatment strategies such as point-of-care tests or introduction of new antibiotics on the progression of AMR [37]. Considering the time required to develop, evaluate and approve new antibiotics and vaccines, our results highlight the urgent need for strategies that can delay the emergence and spread of AMR. Our models, which show the utility of long-term representative surveillance of national and international gonococcal AMR surveillance networks based on isolation and culture of *Neisseria gonorrhoeae* for susceptibility testing [38, 39], can be adapted for use in, and for comparison with, other countries.

## Supplementary Information

**Additional file 1. **GRASP data

**Additional file 2.** Antibiotic prescription

**Additional file 3.** Models

**Additional file 4.** Model fits and predictions

**Additional file 5.** Posterior distributions

## Data Availability

The datasets used and/or analysed during the current study may be available from the UKHSA study authors upon reasonable request to grasp.enquiries@ukhsa.gov.uk or to Hester Allen (hester.allen@ukhsa.gov.uk).
