# Supplementary material for: Projecting the development of antimicrobial resistance in Neisseria gonorrhoeae from antimicrobial surveillance data: a mathematical modelling study
Source: BMC Infect Dis. 2023 Apr 20;23:252. doi: 10.1186/s12879-023-08200-4 (PMC10116452; doi:10.1186/s12879-023-08200-4)

Additional file 1: GRASP data

Resistance data

Data from the GRASP network in the United Kingdom from 2000 to 2018. Susceptibility to antibiotics can be dichotomized into sensitive or resistant using the EUCAST breakpoints (>1 mg/L for azithromycin, >0.06 mg/L for ciprofloxacin, >0.125 mg/L for cefixime, and >0.125 mg/L for ceftriaxone). This data is stratified by sex and sexual orientation in two groups: heterosexual men and women (HMW) and men who have sex with men (MSM).

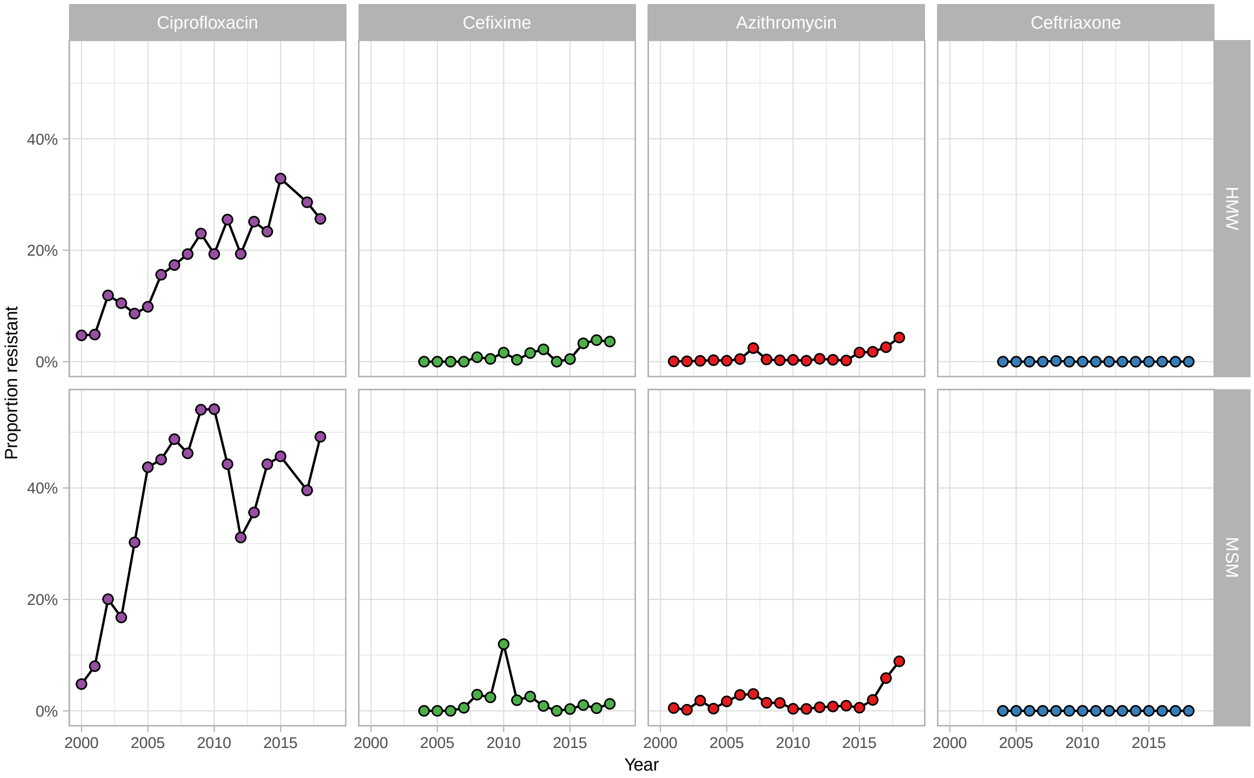

MIC data

For each antibiotic, we also present the evolution of the MIC in time as a stacked area graph, more adapted to observe time trends (the "MIC drift"). For visualization purposes, we group together MIC classes that correspond to resistance according to the EUCAST thresholds.

Ciprofloxacin

The interpretation of ciprofloxacin MIC data is hindered by a technical change in 2010.

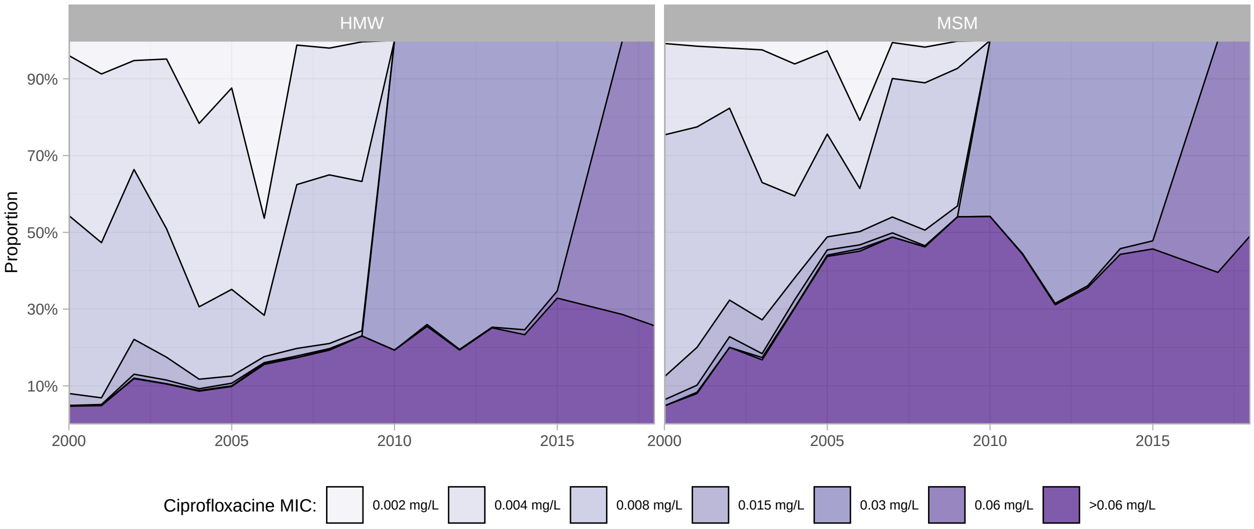

Cefixime

We observe a slight general MIC drift for cefixime for HMW and MSM. This general trend is perturbed by an unusual downward shift of MIC levels in 2006, that remains unexplained. We also observe a temporary upward shift of MIC levels around 2009-2010, mostly among MSM, that can be related to the circulation of an international clone with a novel penA mosaic allele (Unemo et al. 2012).

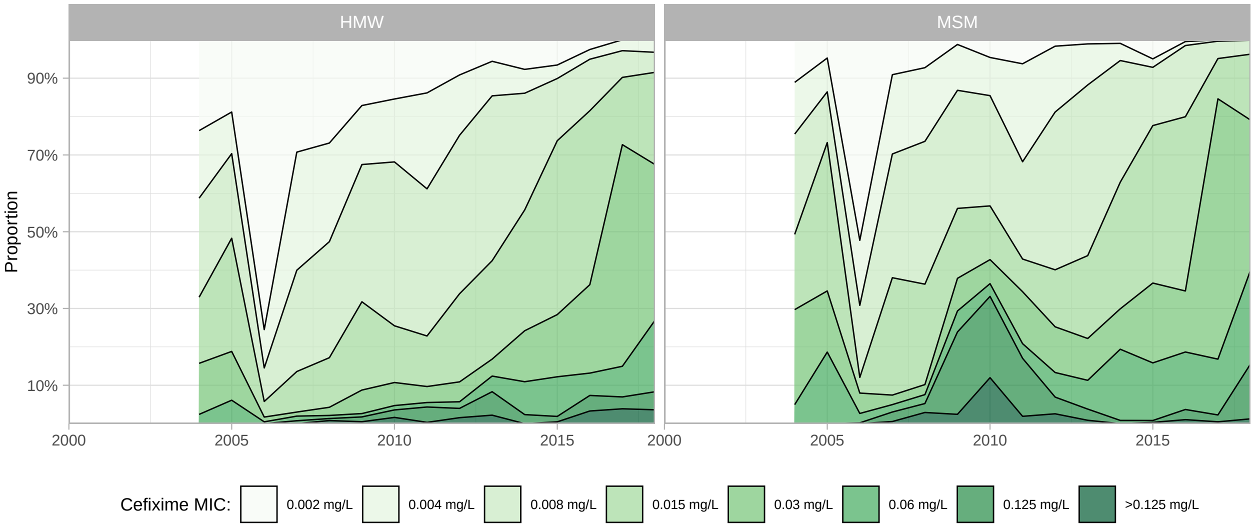

Azithromycin

MIC levels for azithromycin appear relatively stable throughout the study period, with a sudden increase around 2015.

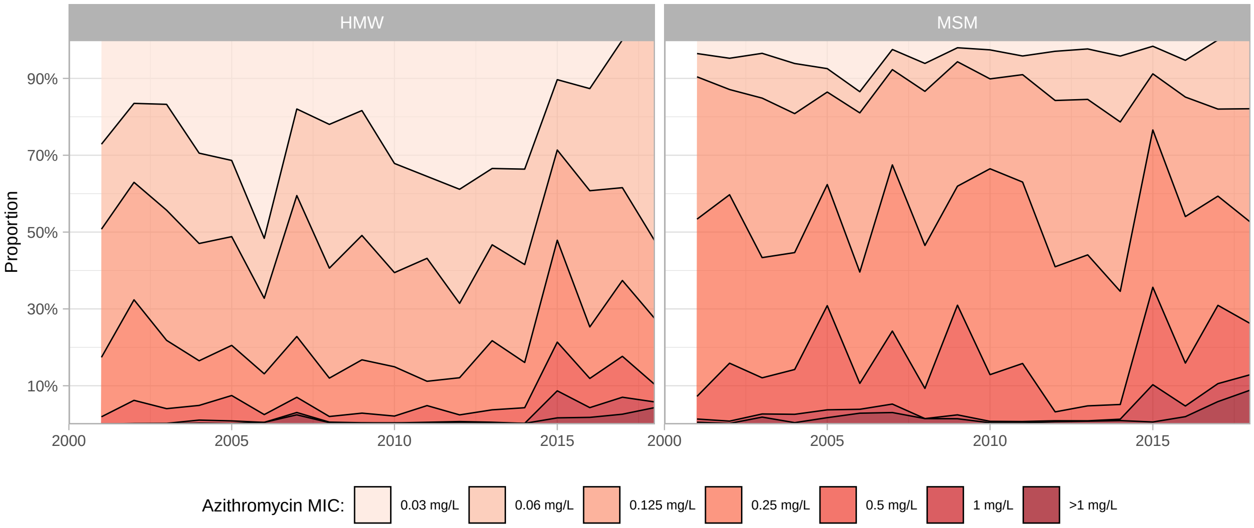

Ceftriaxone

We observe a continuous MIC drift for ceftriaxone in both populations from 2005 on. As for cefixime, we also observe a temporary upward shift of MIC levels around 2009-2010 among MSM, that could be related to the circulation of the international clone.

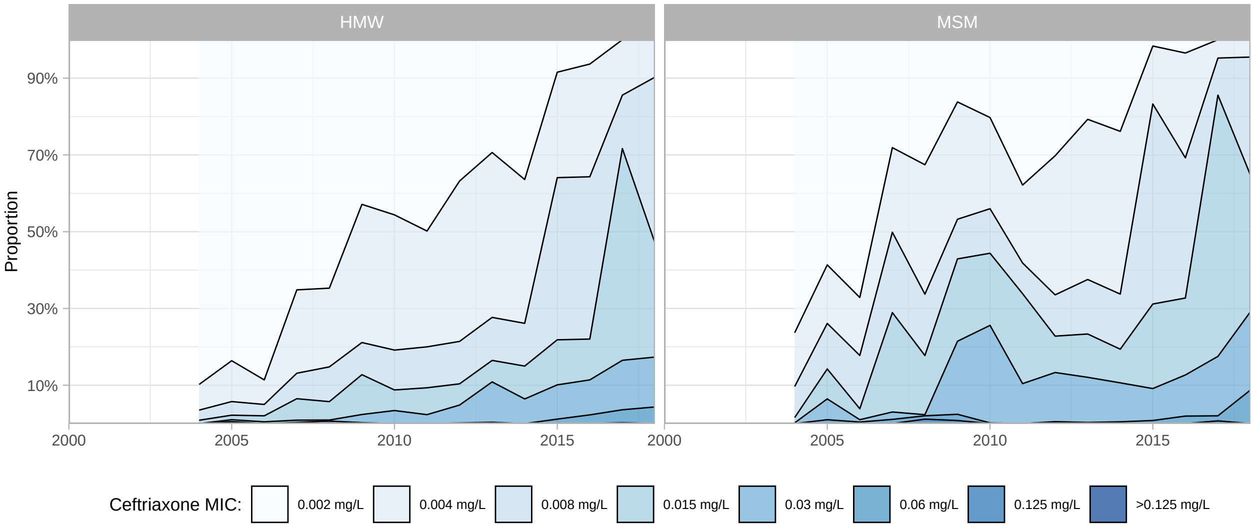

Supplement: Supplementary file 1 — Additional file 1. GRASP data [file 12879_2023_8200_MOESM1_ESM.pdf]
