# Supplementary material for: Projecting the development of antimicrobial resistance in Neisseria gonorrhoeae from antimicrobial surveillance data: a mathematical modelling study
Source: BMC Infect Dis. 2023 Apr 20;23:252. doi: 10.1186/s12879-023-08200-4 (PMC10116452; doi:10.1186/s12879-023-08200-4)

Additional file 2: antibiotic prescription

Antibiotic usage by prescription

Six main combinations of antibiotics prescribed between 2000 and 2018.

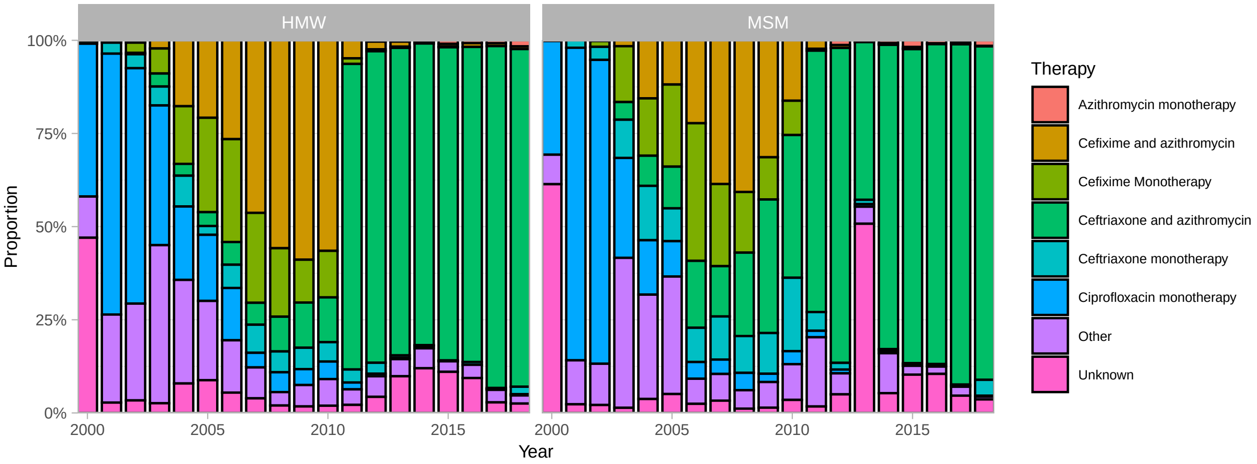

Probability of usage for each of the antibiotics

Data on antibiotic prescription can be transformed into the probability of usage of each of the four antibiotics individually. The inverse triangles at the top indicate the periods of time during which each antibiotic was recommended as a first-line treatment against *N. gonorrhoeae* infection in the UK.

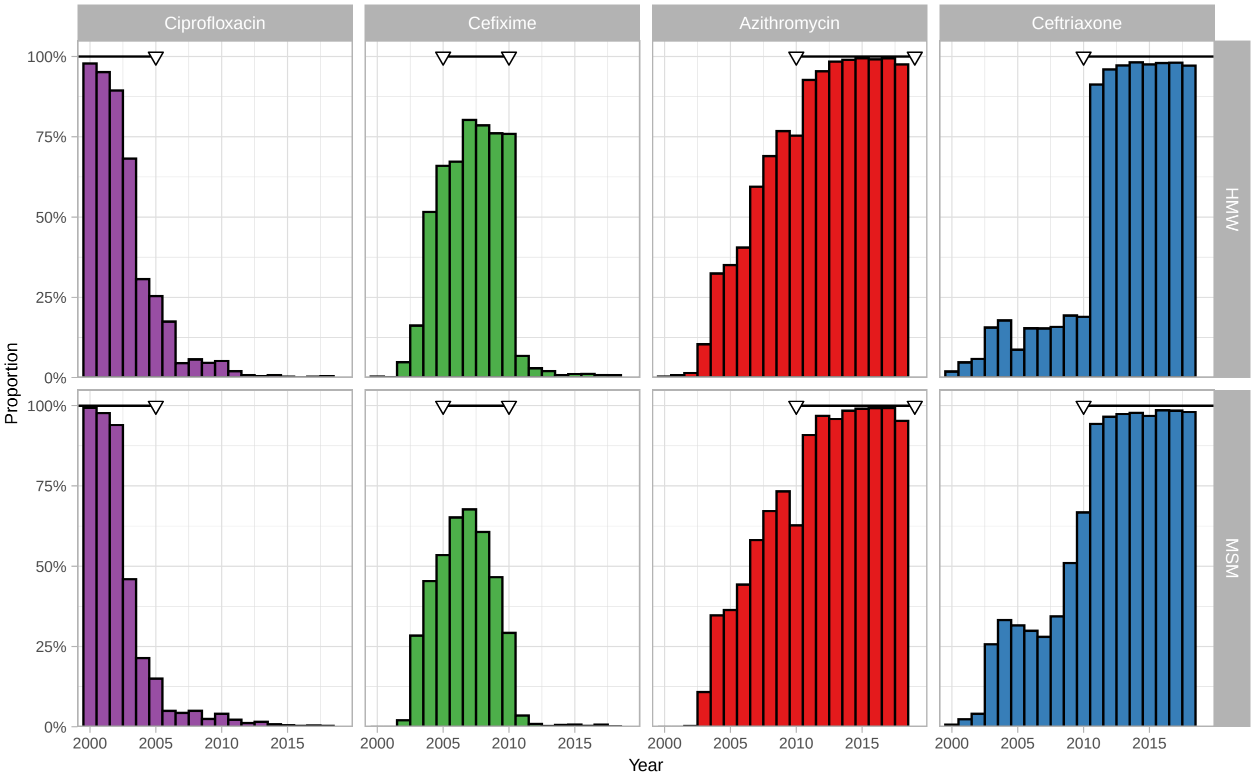

Functional form

One key aspect of modelling the dynamics of resistance in *N. gonorrhoeae* is to account for the selection pressure exerted by antibiotics, and thus to include this information about the probabity of usage of each antibiotic. However, this data in discrete by year, which does not adapt well to ordinary differential equation-based models, that are continuous in nature.

To solve this issue, we chose to use "forcing functions", that is continuous approximations of the data using a closed-form function. We considered using splines or polynomials, but decided on a "two-waves logistic growth and degrowth function" of the following form:

$$p(t) = \frac{\iota_1}{1+\exp(-\delta * (t - t_1 + \xi))} + \frac{1-\iota_1}{1+\exp(-\delta * (t - t_1))} + \frac{\iota_2}{1+\exp(\delta * (t - t_2 - \xi))} + \frac{1-\iota_2}{1+\exp(\delta * (t - t_2))} - 1$$

This seemingly complicated formula is simply a weighted sum of four logistic functions, well-suited to approximate proportions (range 0-1) that increase and decrease over time in response to changing recommendations, sometimes in two waves as for ceftriaxone.

Within this formulation:

- δ is the slope of increase or decrease;
- t_1 and t_2 are median points of introduction or replacement in antibiotic usage;
- (if applicable) if a two-wave introduction is needed, ξ is the delay between the two waves and ι is the intermediate point.

For azithromycin, we fixed t_2 to 2022 to account for the change in recommendations in 2019 and the expected diminution of usage from this date (we assume that the slope of diminution will mirror the slope of increase around 2005). For ciprofloxacin, we fixed t_1 at a very early date as ciprofloxacin was in use at the start of the study period. For the opposite reason, we fixed t_2 at a very late date for ceftriaxone. The other parameters

are estimated from data with least squares. The R code for this procedure is available in `model-dev.R`. This procedure results in the following functions, that are included in the models.

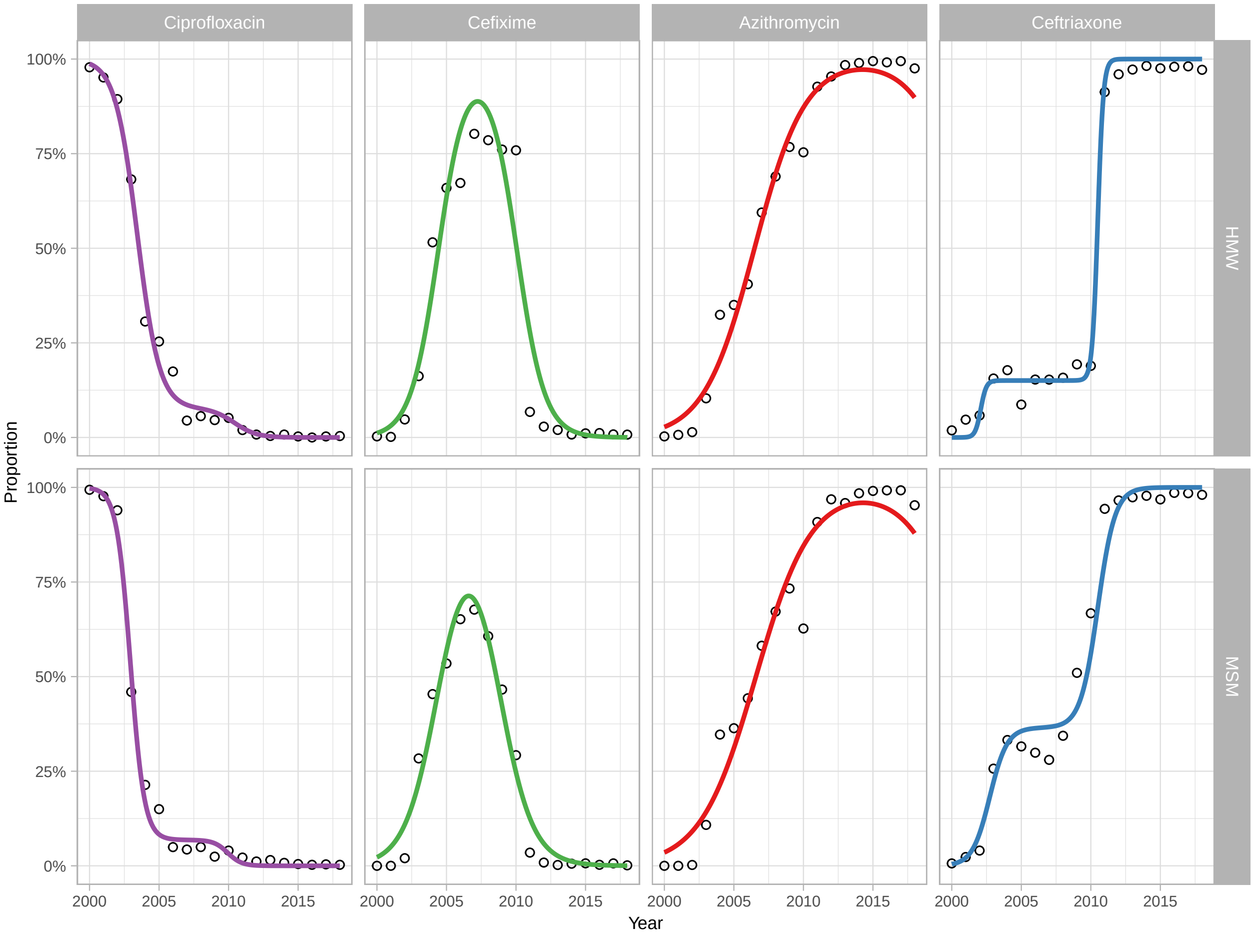

Supplement: Supplementary file 2 — Additional file 2. Antibiotic prescription [file 12879_2023_8200_MOESM2_ESM.pdf]
