# Supplementary material for: Projecting the development of antimicrobial resistance in Neisseria gonorrhoeae from antimicrobial surveillance data: a mathematical modelling study
Source: BMC Infect Dis. 2023 Apr 20;23:252. doi: 10.1186/s12879-023-08200-4 (PMC10116452; doi:10.1186/s12879-023-08200-4)
Supplement: Supplementary file 3 — Additional file 3. Models [file 12879_2023_8200_MOESM3_ESM.pdf]

Additional file 3: models

Single-step model

Model description

The single-step model considers antibiotic resistance as a binary characteristic of *N. gonorrhoeae* with regards to a specific antibiotic. We simply use a SIS-type model (susceptible - infectious - susceptible) with two infectious compartments: I_1 for infected with a wild-type bacteria and I_2 for infected with a resistant bacteria. The model can be expressed with the following system of ordinary differential equations (see also Figure 2 from the paper):

$$\frac{dS}{dt} = -\beta S(I_1 + I_2) + I_1[(1 - p_t)\tau + p_t\tau(1 - \mu) + \nu] + I_2[(1 - p_t)\tau + p_t\tau\epsilon + \nu]$$
$$\frac{dI_1}{dt} = \beta S I_1 - I_1[(1 - p_t)\tau + p_t\tau(1 - \mu) + \nu] - I_1 p\tau\mu$$
$$\frac{dI_2}{dt} = \beta S I_2 - I_2[(1 - p_t)\tau + p_t\tau\epsilon + \nu]$$

Susceptible individuals become infected with either type of infection $k \in \{1, 2\}$ according to a force of infection $\beta S I_k$. Infected individuals recover spontaneously at rate ν, or through treatment. Here we introduce prescription data under the form of the forcing function $p(t)$, representing the probability that a prescription at time t actually includes the antibiotic of interest. If the antibiotic of interest is prescribed (probability $p(t)$) then the effect of treatment differs according to the resistance status. Infections by wild-type bacteria (compartment I_1) can either recover (with probability $1 - \mu$) or develop resistance in one single step (with probability μ). For infections by resistant-type bacteria (compartment I_2) recovery through treatment occurs with a lower efficacy $\epsilon \in [0, 1]$. If another antibiotic is prescribed (probability $1 - p(t)$), recovery through treatment occurs at the same rate τ for compartments I_1 and I_2.

Reparameterization of β

We assume an initial situation where the prevalence of *N. gonorrhoeae* is at endemic equilibrium. To do that, we reparameterize β as a function of the other parameters, and of the prevalence at endemic equilibrium I^*.

In a first step, we retrieve the formula of $\mathcal{R}_0$ in the single-step model using the next generation matrix method (Diekmann et al, 1990; Van den Driessche and Watmough, 2002). This implies the computation of the infection matrix F:

$$F = \begin{bmatrix} \beta & 0 \\ 0 & \beta \end{bmatrix},$$

and of the migration matrix V:

$$V = \begin{bmatrix} \tau + \nu & 0 \\ -\mu\tau & \tau\epsilon + \nu \end{bmatrix}.$$

We can then find $\mathcal{R}_0$ as the largest eigenvalue of the next generation matrix FV^{-1}, which results in:

$$\mathcal{R}_0 = \frac{\beta}{\nu + \tau}.$$

In a second step, we consider that the prevalence at endemic equilibrium I^* is related to $\mathcal{R}_0$ through:

$$I^* = 1 - \frac{1}{\mathcal{R}_0}.$$

We can now express the transmission parameter β as function of I^* and the other parameters:

$$\beta = \frac{\nu + \tau}{1 - I^*}.$$

We thus replace β by this formula in the model, ensuring that the model will start at endemic equilibrium. From now, we treat I^* as a parameter, with a normal prior distribution based on estimates of prevalence ranging between 0.16% and 0.38% in HMW and between 1.19% and 2.79% in MSM (Fingerhuth et al. 2016).

Joint model and inference

In total, this model describes the population-level dynamics of resistance development in relation to antibiotic usage in a population with five parameters: $\{I^*, \nu, \tau, \mu, \epsilon\}$. In addition, we consider the initial conditions of resistance in the population, that we treat as another parameter ρ.

To support identifiability, we expand this framework to jointly model the development of resistance in HMW ($i = 1$) and MSM ($i = 2$). This takes advantage of the fact that some parameters may be assume to have common values in these two groups, such as the recovery rate ν, the probability of mutation given treatment μ and the treatment efficacy ϵ. The other parameters are left independent (τ_i, I_i^* and rho_i) between the two groups, except for the constraint that τ must be higher for MSM than for HMW (implemented using the `ordered` data type in Stan).

We estimate all parameters by fitting the model to resistance data using following likelihood:

$$\Pr(\text{data}|I_i^*, \nu, \tau_i, \mu, \epsilon, \rho_i, \kappa) = \prod_{t,i} \text{beta-binomial}\left(n_{t,i}, \Bbbk_{t,i} \middle| \kappa \frac{I_{2,i}(t)}{I_{1,i}(t) + I_{2,i}(t)}, \kappa\left(1 - \frac{I_{2,i}(t)}{I_{1,i}(t) + I_{2,i}(t)}\right)\right)$$

where $\Bbbk_{t,i}$ is the number of isolates with resistance at time t in population i, $n_{t,i}$ is the sample size, and κ is an overdispersion parameter.

Prior distributions

We selected the following weakly-informative prior distributions:

$$\nu \sim \text{exponential}(1)$$

$$\tau_i \sim \text{log-normal}(0, 1)$$
$$\mu \sim \text{beta}(1, 1000)$$
$$\epsilon \sim \text{beta}(1, 1)$$
$$\rho_i \sim \text{beta}(1, 1)$$

$$\kappa \sim 2 + \text{exponential}(0.01)$$

These choices were made by considering the range of outcomes implied in prior predictive checks (Gabry et al. 2019). Indeed, the priors implied that the resistance levels can vary basically between 0 and 100% over the period 2010 to 2050. The following figure shows 2,000 trajectories implied by the chosen priors:

```
load("models/samples_2022-05-17/S_binary_grasp_azithro_2022-05-17.Rdata")
plot_summary_prior(SIM_binary_grasp_azithro,lim=2050,colmic = "Greys")
```

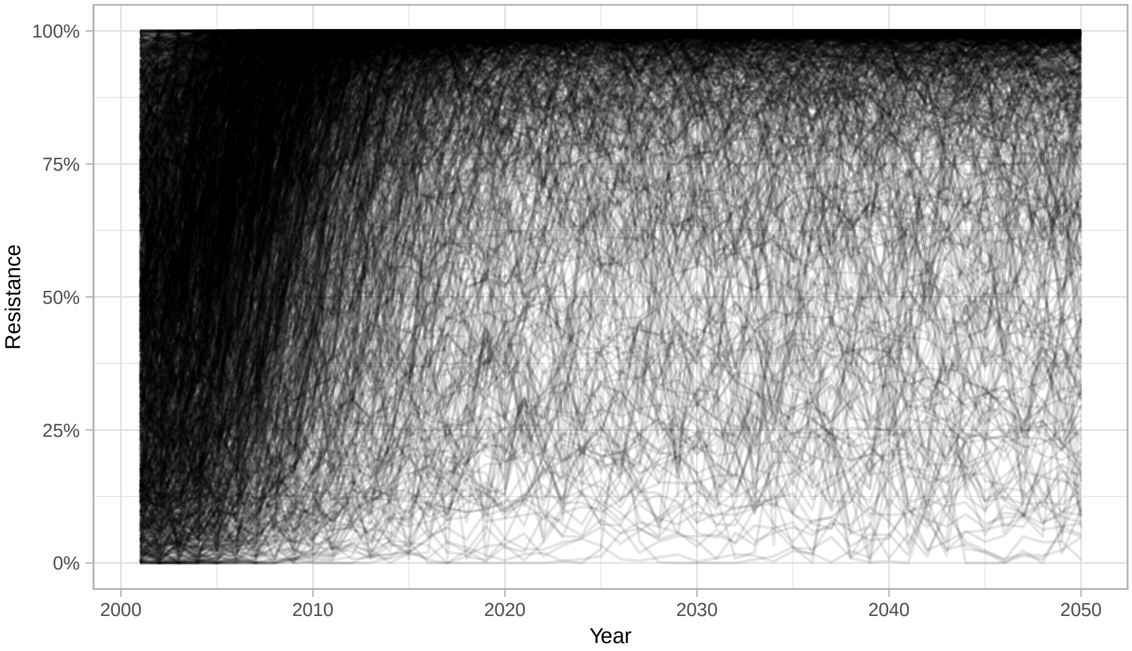

Choosing a "flat" prior on μ (e.g. beta$(1,1)$) would have resulted in greatly favoring scenarios with a very quick increase of resistance, as shown in the following figure:

```
load("models/samples_2022-05-17/S_binary_grasp_azithro_flat_2022-05-17.Rdata")
plot_summary_prior(SIM_binary_grasp_azithro_flat,lim=2050,colmic = "Greys")
```

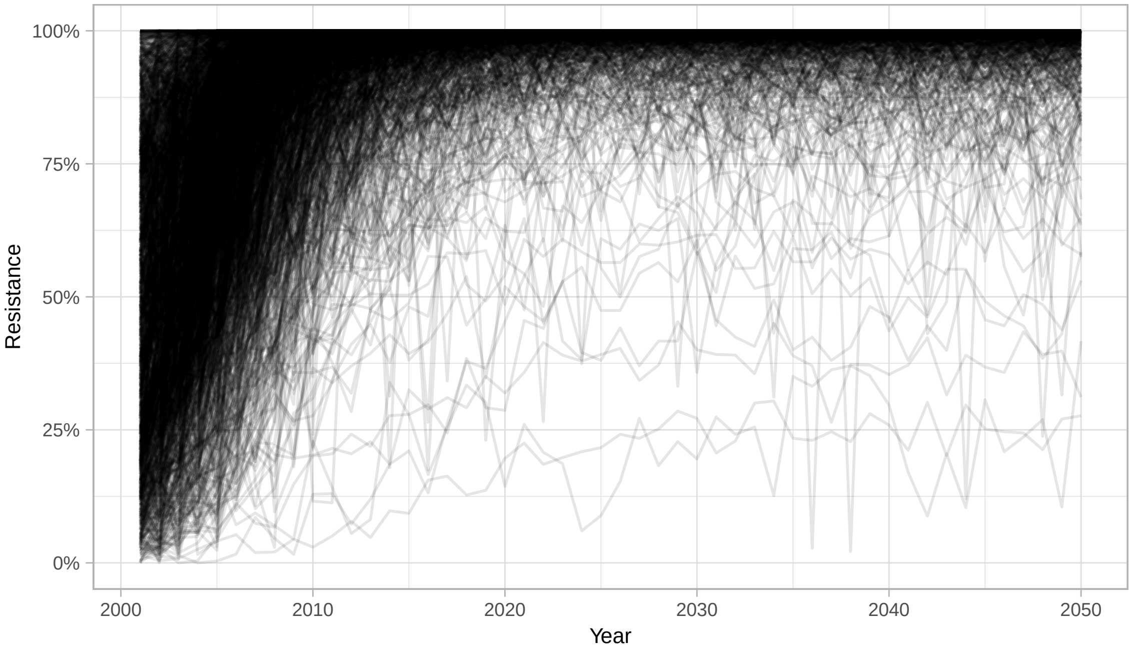

We argue that using a prior on μ that favors smaller values allows for a wider range of scenarios, and is thus more adequate that the "flat" priors.

Implementation

We implemented this model in Stan, and conduct parameter inference with Hamiltonian Monte Carlo using Stan default NUTS algorithm. We assessed the quality of the inference by applying diagnosis tools (divergent transitions, tree depth, E-BFMI), and by observing the trace plots and the posterior predictive check plots.

The model code is available in `models/binary_model.stan`. The R function to format the data and actually run inference is available in `fit_binary_model.R`. The application to GRASP data is done in `main-binary.R`.

Multi-step model

Model description

We consider an alternative multi-step model that treats resistance acquisition as a multi-step, cumulative process. The model follows a similar structure as the single-step model but includes multiple infected compartments $\{I_1, \ldots, I_k\}$ instead of two. These K compartments represent increasing levels of antibiotic resistance and correspond to the K MIC classes reported in GRASP (e.g., $K = 8$ in the case of ceftriaxone):

$$\frac{dS}{dt} = -\beta S \sum_{k=1}^{K} I_k + p_t \tau (1-\mu) \sum_{k=1}^{K-1} \epsilon_k I_k + p_t \tau \epsilon_K I_K + (1-p_t)\tau \sum_{k=1}^{K} I_k + \nu \sum_{k=1}^{K} I_k$$

$$\frac{dI_1}{dt} = \beta S I_1 - p_t \tau \mu I_1 - p_t \tau (1-\mu)\epsilon_1 I_1 - (1-p_t)\tau I_1 - \nu I1$$

$$\frac{dI_{k \in 2..K-1}}{dt} = \beta S I_k + p_t \tau \mu I_{k-1} - p_t \tau \mu I_k - p_t \tau (1-\mu)\epsilon_k I_k - (1-p_t)\tau I_k - \nu I_k;$$

$$\frac{dI_K}{dt} = \beta S I_k + p_t \tau \mu I_{K-1} - p_t \tau \epsilon_K I_K - (1-p_t)\tau I_K - \nu I_K;$$

This model relies upon two central assumptions. First, the probability of developing one more step of resistance upon treatment μ is the same for every class. Second, increasing levels of AMR lead to a linear decrease in treatment efficacy, with a linear interpolation between ϵ_1 (fixed to 100%) and $\epsilon_K \in [0, 1]$ (estimated):

$$\epsilon_k = 1 - \frac{k-1}{(K-1)(1-\epsilon_K)}$$

A progressive decrease of treatment efficacy with MIC is compatible with the pharmacodynamical concept of a "period with the free drug level above MIC' ' necessary to achieve treatment efficacy [**?**]. This second model is also based on five parameters: $\{\beta, \nu, \tau, \mu, \epsilon_K\}$.

Joint model and inference

We use the same reparameterization of β in terms of the other parameters and the prevalence at endemic equilibrium I^*. The initial conditions of resistance are now modelled by a simplex vector of K elements ρ. We also jointly model the growth of MIC in HMW ($i = 1$) and MSM ($i = 2$), with the same common parameters ν, μ and ϵ_K.

We estimate all parameters by fitting the model to resistance data using following likelihood:

$$\Pr(\text{data}|I_i^*, \nu, \tau_i, \mu, \epsilon_K, \rho_i, \phi) = \prod_{t,i} \text{dirichlet-multinomial}\left(\Bbbk_{t,i} \middle| \phi \frac{I_{k,i}(t)}{\sum_k I_{k,i}(t)}\right)$$

where $\Bbbk_{t,i}$ is the number of isolates within each MIC class at time t and in population i, and ϕ is an overdispersion parameter.

Prior distributions

We selected the following weakly-informative prior distributions:

$$\nu \sim \text{exponential}(1)$$

$$\tau_i \sim \text{log-normal}(0, 1)$$

$$\mu \sim \text{beta}(1, 100)$$

$$\epsilon_K \sim \text{beta}(1,1)$$
$$\rho_i \sim \text{dirichlet}(1,...,1)$$
$$\phi \sim \text{exponential}(0.01)$$

The slightly larger prior on μ is justified by the fact that the probability of developing one more step of resistance upon treatment has to be larger than the probability of developing full resistance directly. We validate our choice of priors with prior predictive checks:

```
load("models/samples_2022-05-17/S_multistep_grasp_azithro_2022-05-17.Rdata")
plot_summary_prior2(SIM_multistep_grasp_azithro,lim=2050,colmic = "Greys")
```

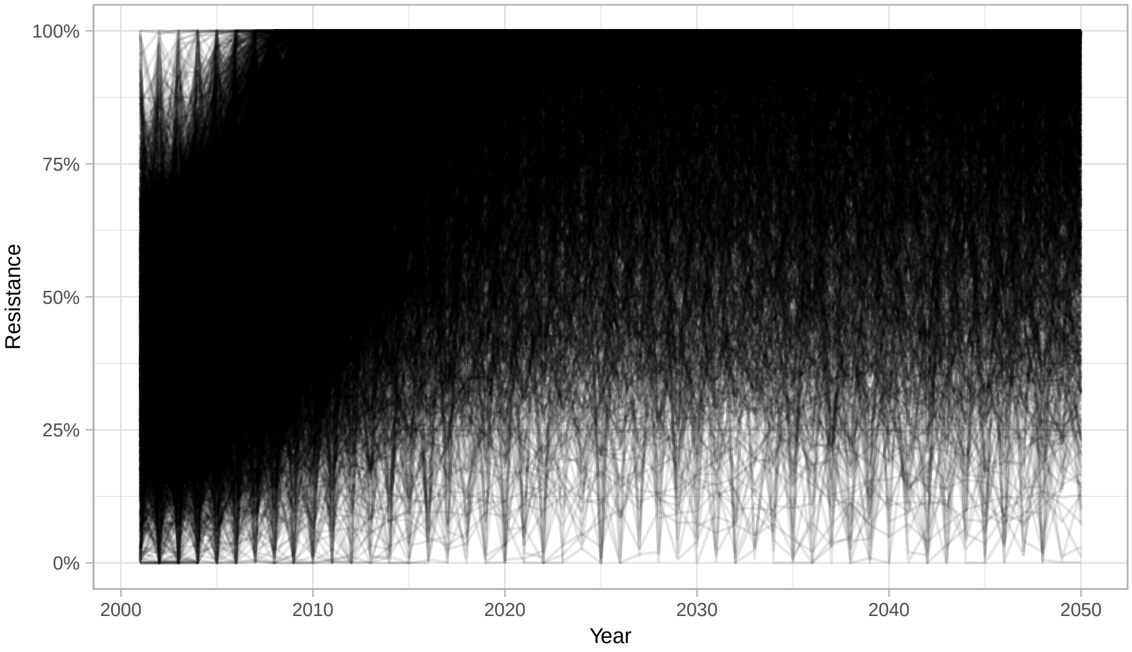

We see that the chosen priors allow for a large range of scenarios.

Implementation

We also implemented this model in Stan and use the same inference procedure. The model code is available in `models/multistep_model.stan`. The R function to format the data and actually run inference is available in `fit_multistep_model.R`. The application to GRASP data is done in `main-multistep.R`.

Sensitivity analyses

Removing data from 2009-2010

We considered a sensitivity analysis where data from 2009 and 2010 was removed. The objective was to evaluate the impact of the temporary rise of the number of observed cases of high resistance in these years, that can be attributed to the international circulation of a multi-drug resistance clone. This was simply implemented by removing observations from 2009 and 2012 from the data.

Increasing prevalence

Another sensitivity analysis aimed at relaxing our hypotheses regarding the prevalence of *N. gonorrhoeae* infections. In the main analysis, we assumed an initial situation where the prevalence of *N. gonorrhoeae* is at endemic equilibrium, and reparameterized β as a function of the other parameters. We now consider a situation where the prevalence of *N. gonorrhoeae* is steadily rising over time. This is implemented by adding a

time-dependent component to β, so that it starts at the pre-specified prevalence, and rises linearly throughout the years:

$$\beta'(t) = \beta \times (1 + \zeta t)$$

where t is the number of years since initiation. This is implemented in models `binary_modelB.stan` and `multistep_modelB.stan`. We considered three scenarios with the parameter ζ fixed to 0.001, 0.005 or 0.01, corresponding to increasing slopes in the rise of prevalence.

References

Diekmann, O., Heesterbeek, J. A. P., & Metz, J. A. (1990). On the definition and the computation of the basic reproduction ratio R 0 in models for infectious diseases in heterogeneous populations. Journal of mathematical biology, 28(4), 365-382.

Fingerhuth, S. M., Bonhoeffer, S., Low, N., & Althaus, C. L. (2016). Antibiotic-resistant Neisseria gonorrhoeae spread faster with more treatment, not more sexual partners. PLoS pathogens, 12(5), e1005611.

Gabry, J., Simpson, D., Vehtari, A., Betancourt, M., & Gelman, A. (2019). Visualization in Bayesian workflow. Journal of the Royal Statistical Society: Series A (Statistics in Society), 182(2), 389-402.

Van den Driessche, P., & Watmough, J. (2002). Reproduction numbers and sub-threshold endemic equilibria for compartmental models of disease transmission. Mathematical biosciences, 180(1-2), 29-48.


```r
# rmarkdown::render("S3_model.R")
```
