# Supplementary material for: Projecting the development of antimicrobial resistance in Neisseria gonorrhoeae from antimicrobial surveillance data: a mathematical modelling study
Source: BMC Infect Dis. 2023 Apr 20;23:252. doi: 10.1186/s12879-023-08200-4 (PMC10116452; doi:10.1186/s12879-023-08200-4)
Supplement: Supplementary file 4 — Additional file 4. Model fits and predictions [file 12879_2023_8200_MOESM4_ESM.pdf]

Additional file 4: model fit and predictions

We present here the full results from the application of the single-step and multi-step models to data from GRASP. All the posterior samples used for this work are available in `models`. Files bigger than 50 Mb were split into several `.7z` archives for storing on github.

For each model and each antibiotic, we present a figure summarizing different aspects of the model results. Panel (A) shows the model fit; panel (B) is the evolution of treatment usage with time; panel (C) is the model-based projection of NG antimicrobial resistance (using the appropriate EUCAST threshold) until 2030; panel (D) is the projection of NG prevalence if influenced only by resistance levels; and (E) is the evolution of the probability of reaching the 5%, 10% and 20% thresholds. We present results from the single-step model, and when applicable (that is, for all antibiotics except ciprofloxacin), results from the multi-step model.

Ciprofloxacin

Single-step model

```
plot_summary(S_binary_grasp_ciprofloxacin,lim=2031,legend.pos=c(.8,.3),colmic = "Purples")
```

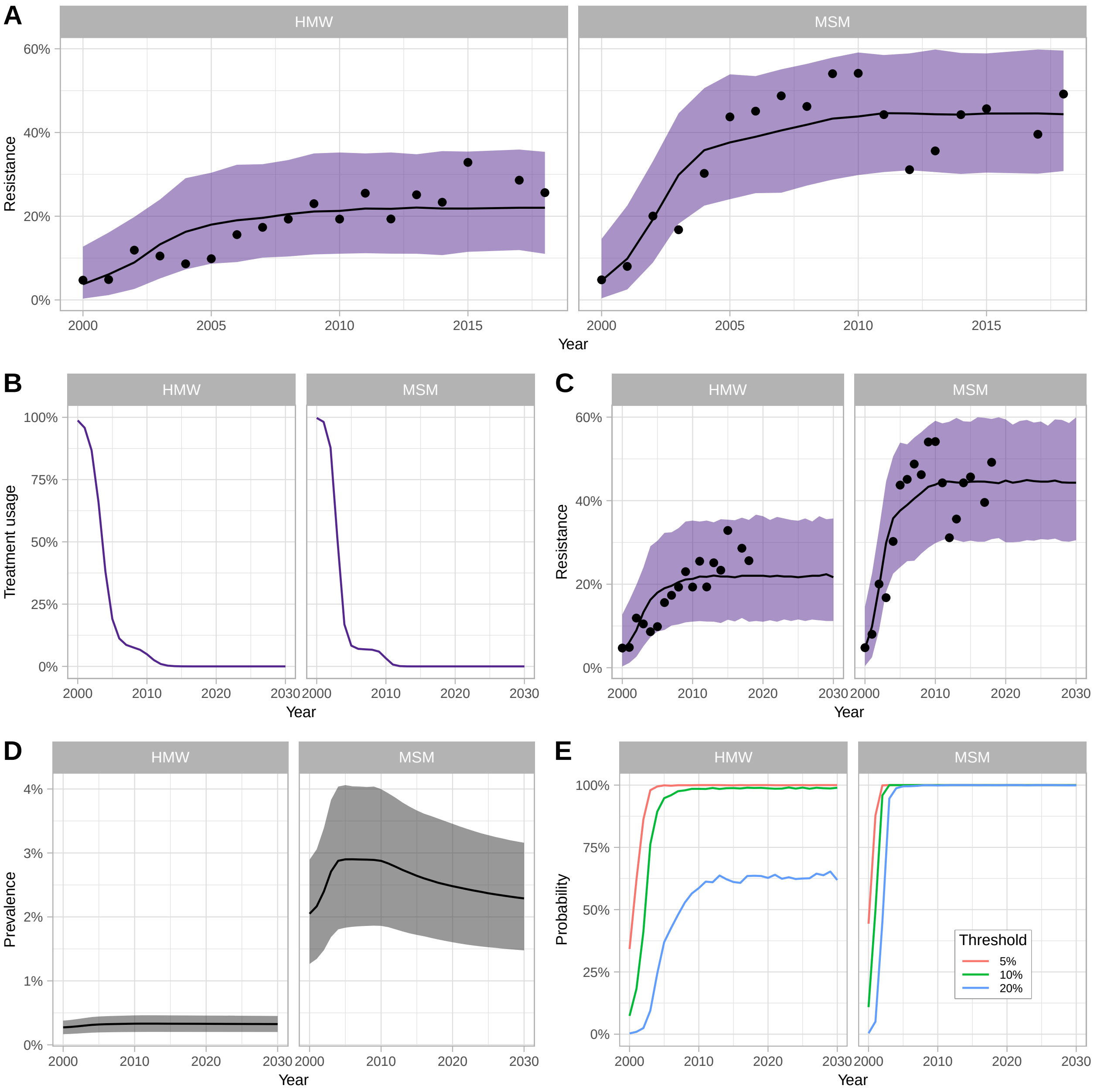

Multi-step model

Not done because of the lack of detail on MIC after 2009 in the data

Cefixime

Single-step model

```
plot_summary(S_binary_grasp_cefixime,lim=2031,legend.pos=c(.8,.7),colmic = "Greens")
```

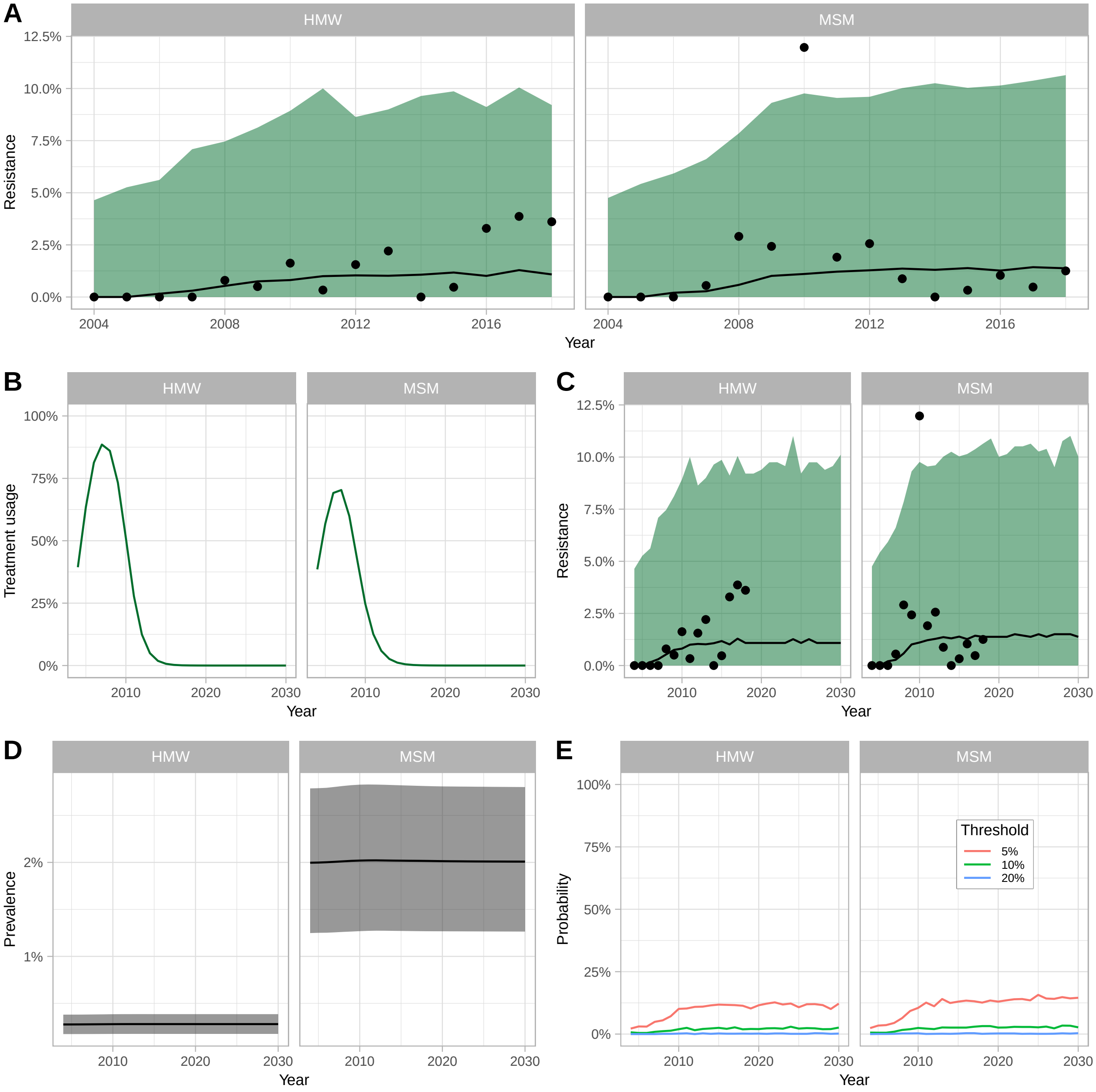

Multi-step model

```
plot_summary2(S_multistep_grasp_cefixime,lim=2031,legend.pos=c(.8,.7),colmic = "Greens")
```

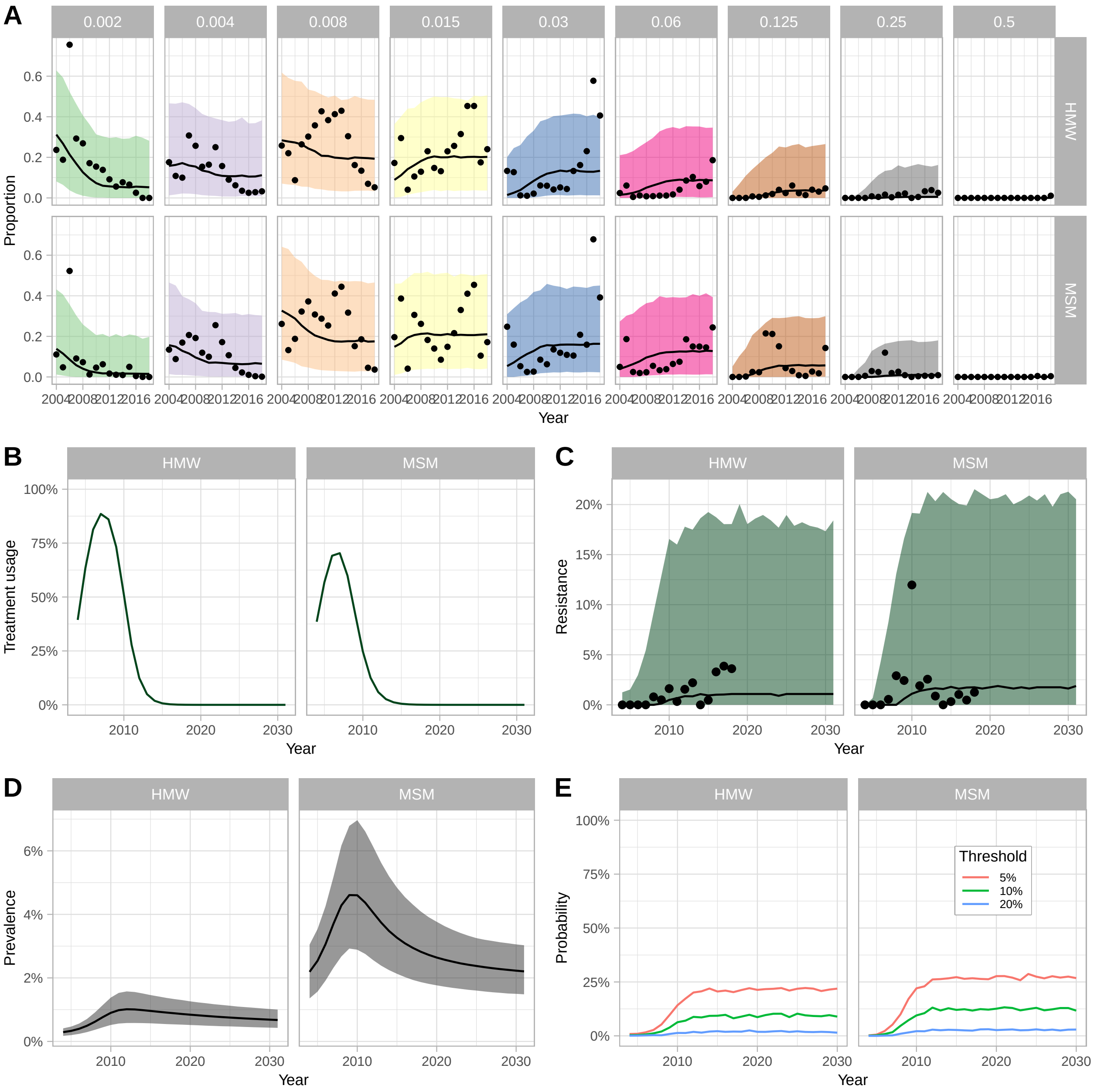

Azithromycin

Single-step model

```
plot_summary(S_binary_grasp_azithro,lim=2031,legend.pos=c(.4,.3),colmic = "Reds")
```

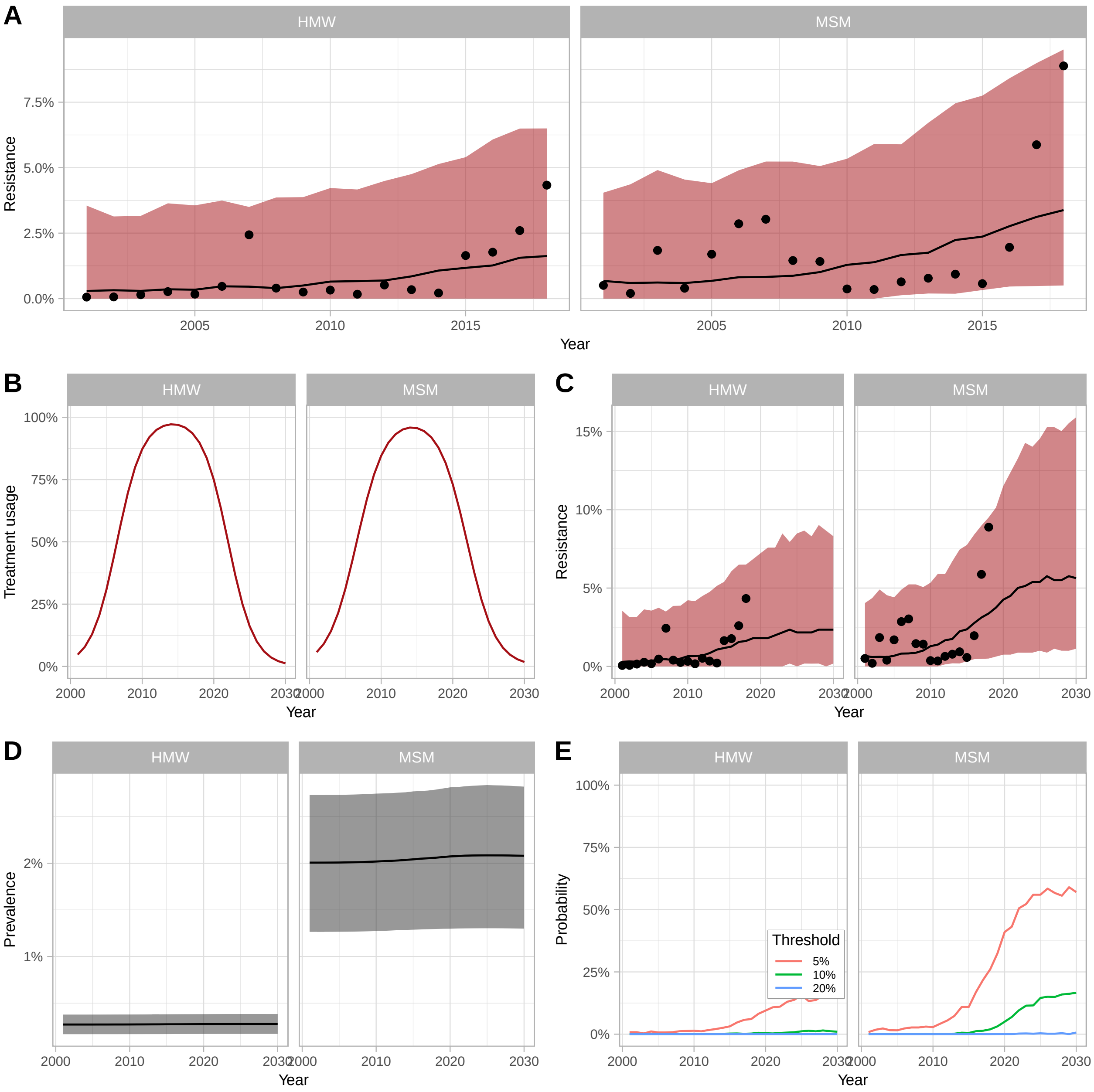

Multi-step model

```
plot_summary2(S_multistep_grasp_azithro,lim=2031,legend.pos=c(.4,.3),colmic = "Reds")
```

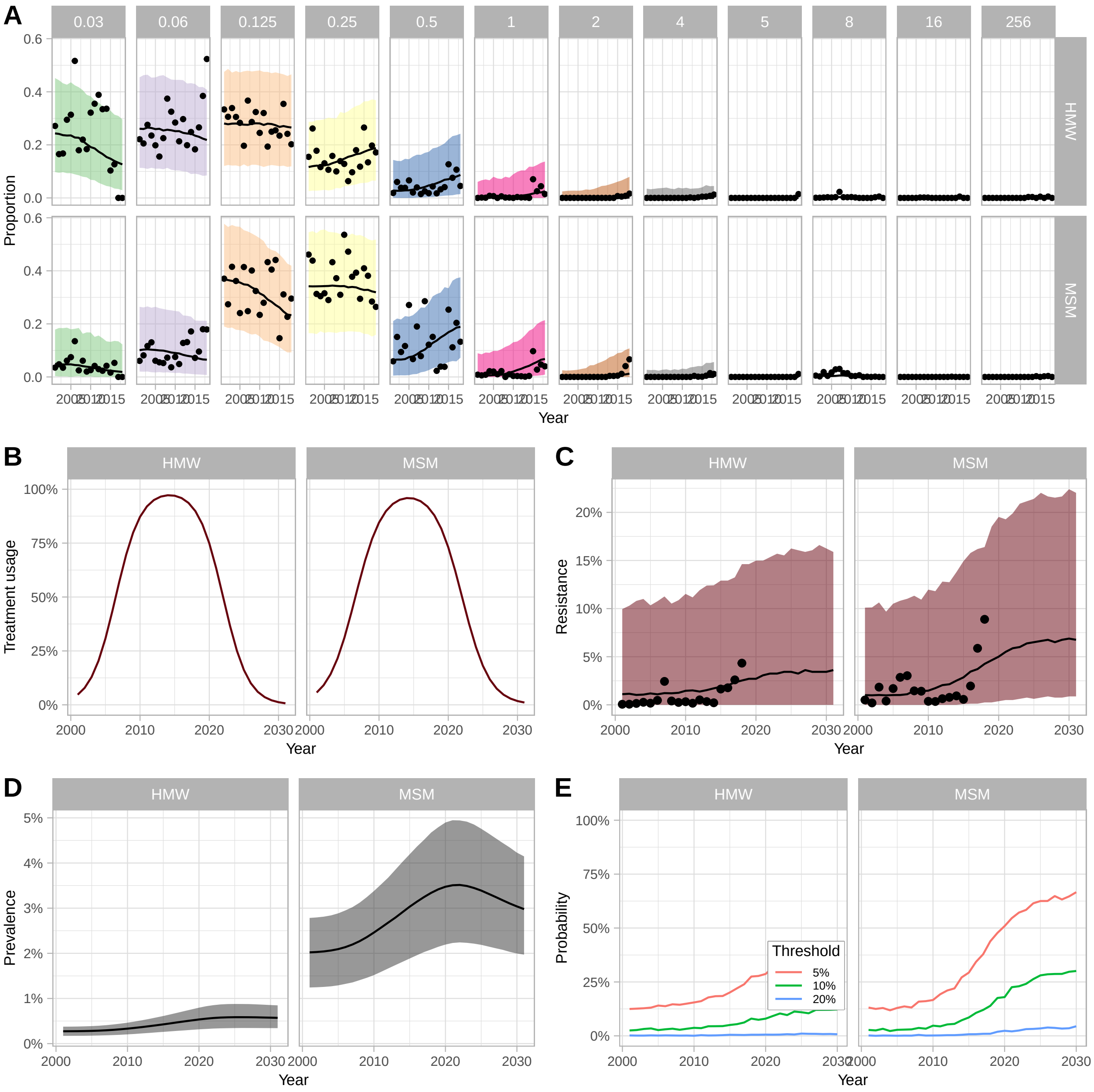

Ceftriaxone

Single-step model

```
plot_summary(S_binary_grasp_ceftriaxone,lim=2031,legend.pos=c(.8,.7),colmic = "Blues")
```

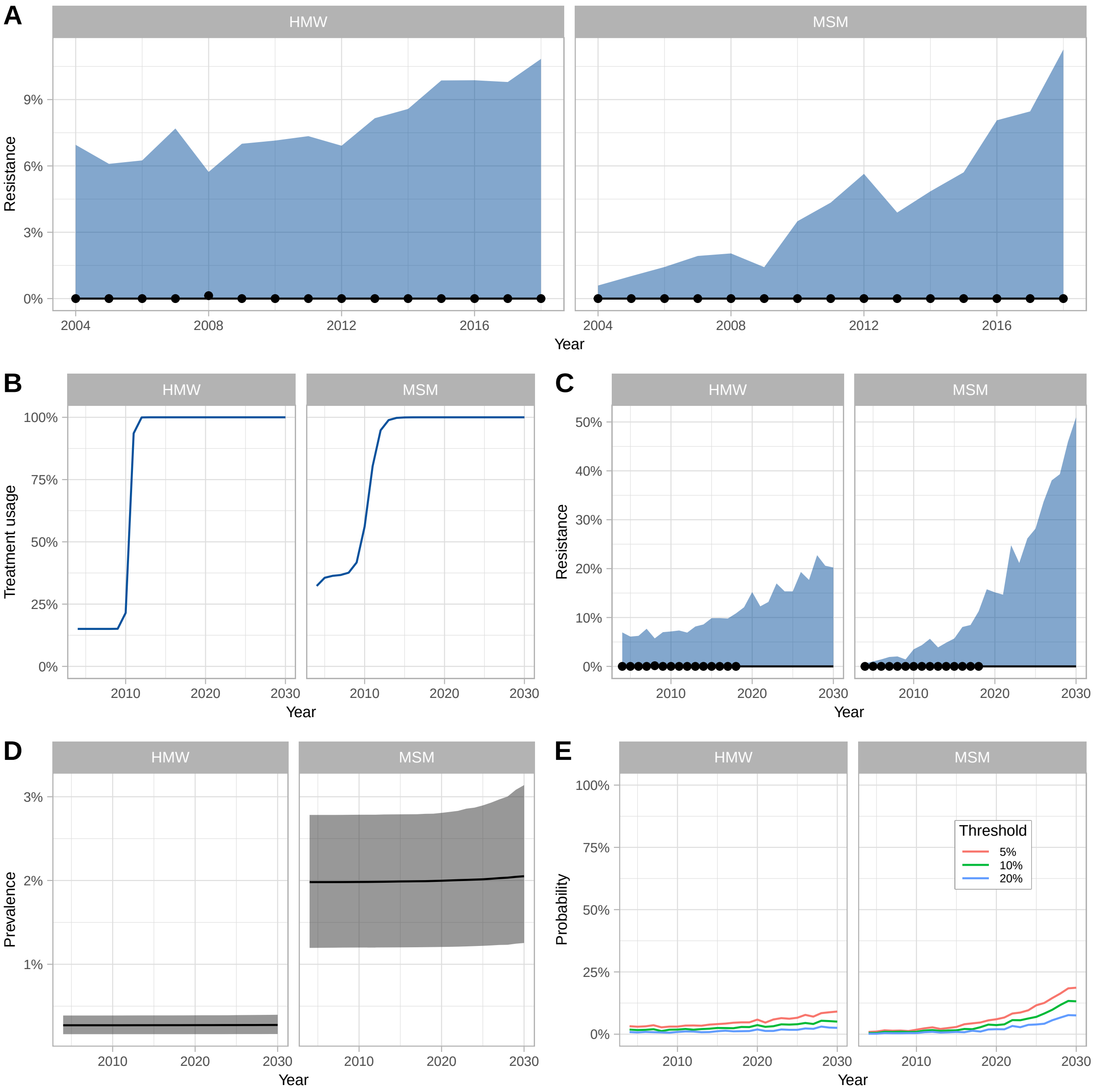

Multi-step model

With all data from 2004 to 2018

```
plot_summary2(S_multistep_grasp_ceftriaxone,lim=2031,legend.pos=c(.8,.7),colmic = "Blues")
```

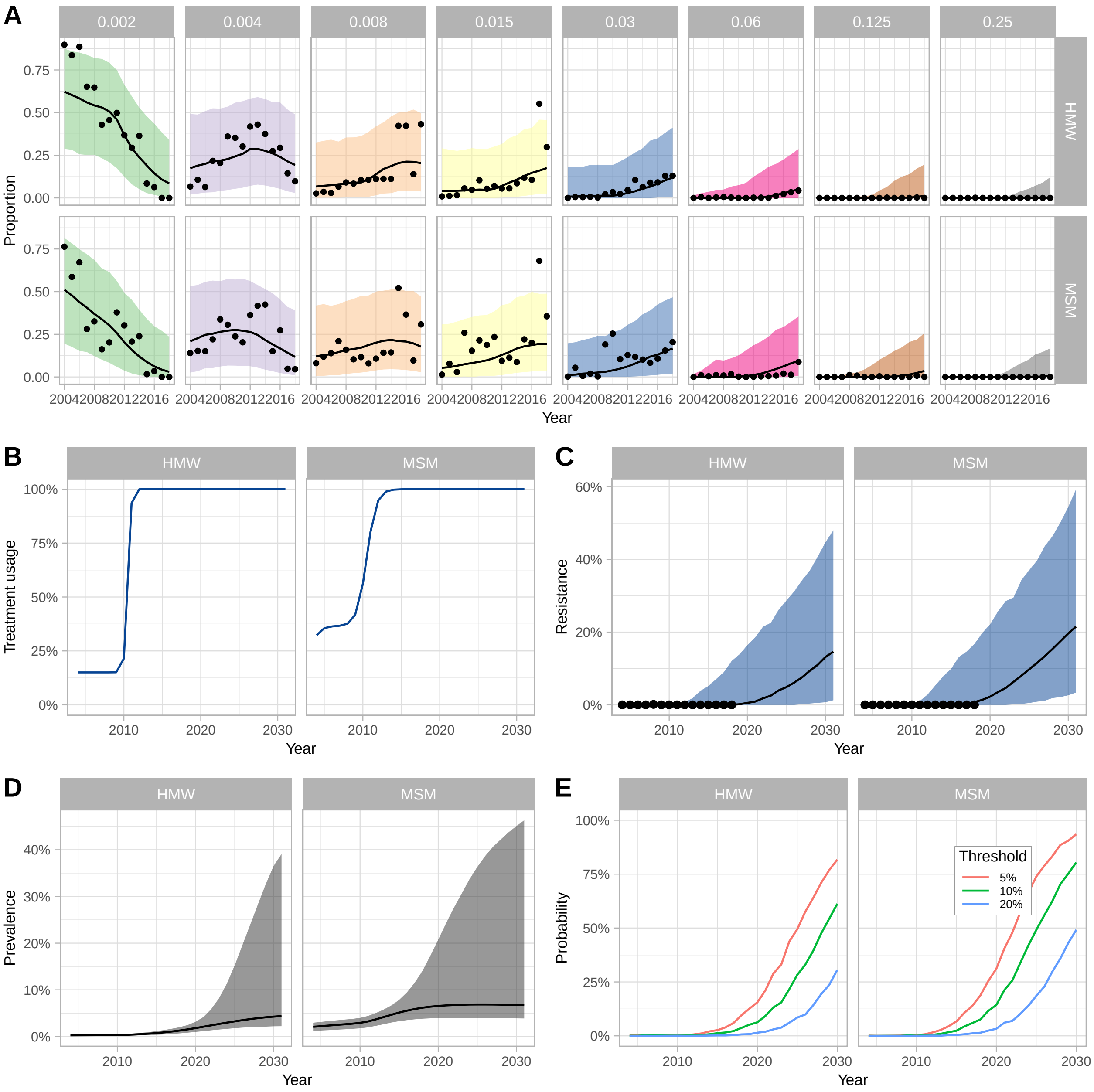

Sensitivity analysis removing data from 2009-2010

```
plot_summary2(S_multistep_grasp_ceftriaxone_ss1,lim=2031,legend.pos=c(.8,.7),colmic = "Blues")
```

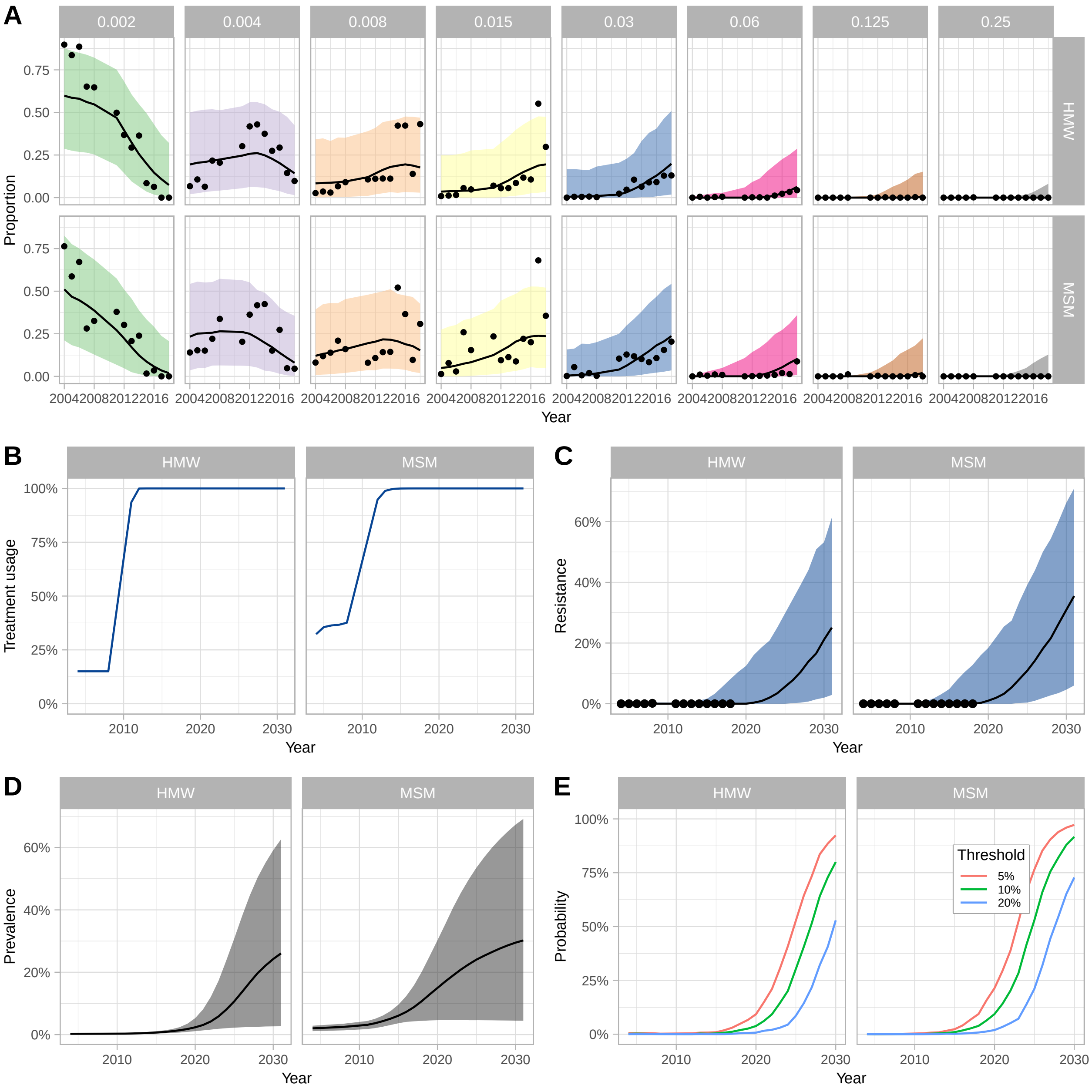

Sensitivity analysis with increasing transmission

Yearly increase of β by 1.001

```
plot_summary2(S_multistep_grasp_ceftriaxone_incprev001,lim=2031,legend.pos=c(.8,.7),colmic = "Blues")
```

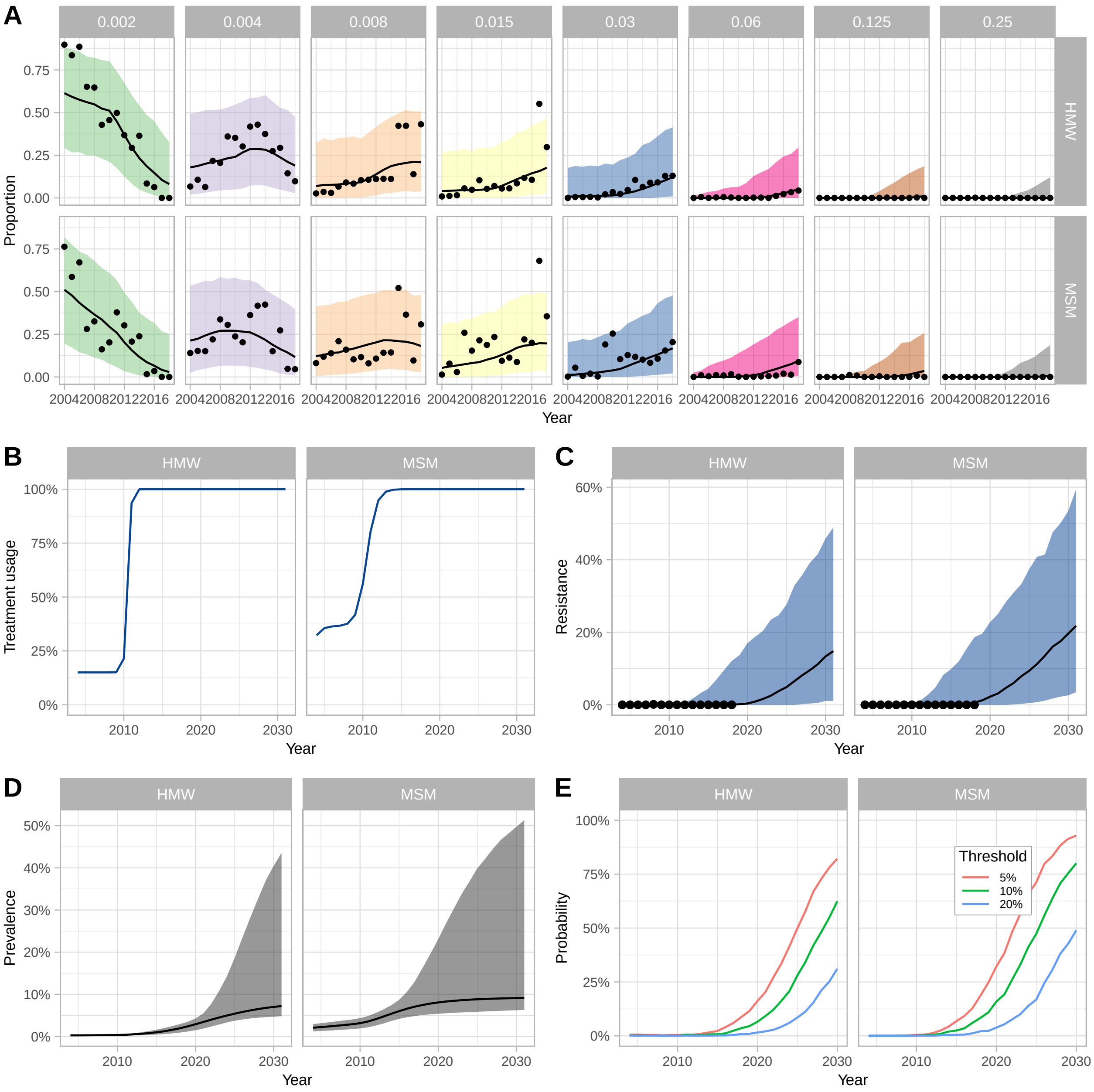

Yearly increase of β by 1.005

```
plot_summary2(S_multistep_grasp_ceftriaxone_incprev005,lim=2031,legend.pos=c(.8,.7),colmic = "Blues")
```

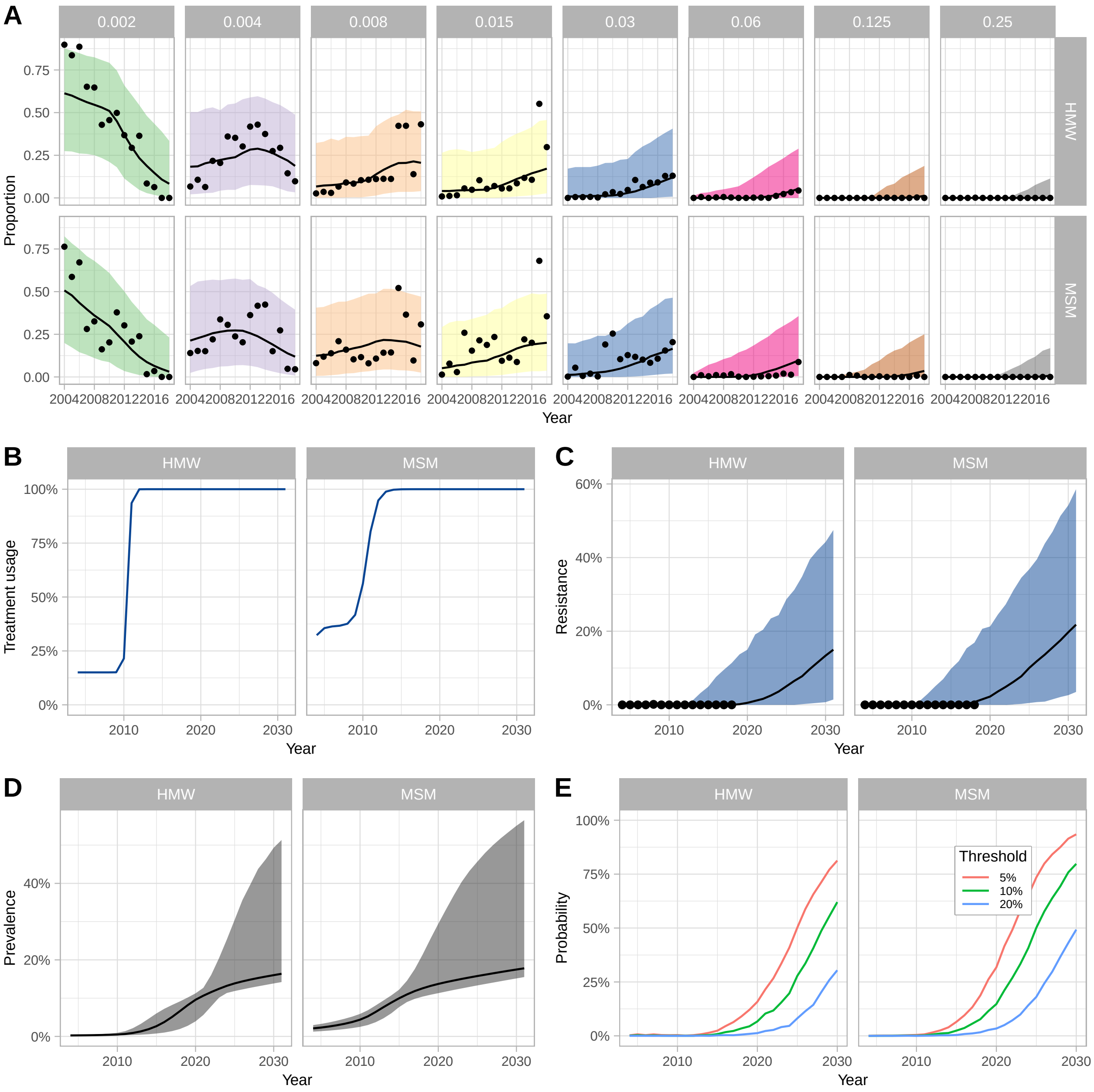

Yearly increase of β by 1.01

```
plot_summary2(S_multistep_grasp_ceftriaxone_incprev01,lim=2031,legend.pos=c(.8,.7),colmic = "Blues")
```

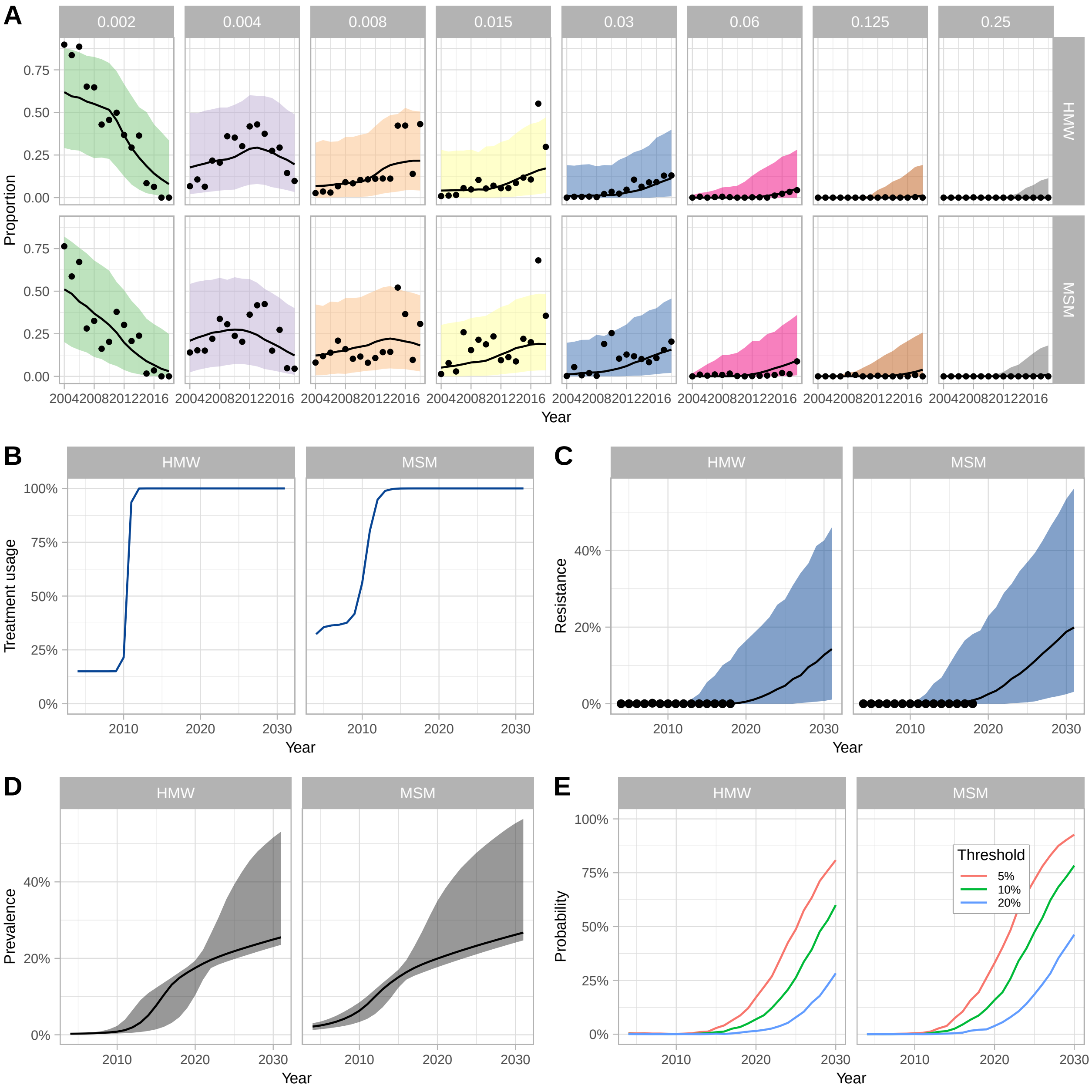


```
# rmarkdown::render("S4_model_fits_predictions.R")
```
