# Supplementary material for: Projecting the development of antimicrobial resistance in Neisseria gonorrhoeae from antimicrobial surveillance data: a mathematical modelling study
Source: BMC Infect Dis. 2023 Apr 20;23:252. doi: 10.1186/s12879-023-08200-4 (PMC10116452; doi:10.1186/s12879-023-08200-4)
Supplement: Supplementary file 5 — Additional file 5. Posterior distributions [file 12879_2023_8200_MOESM5_ESM.pdf]

Additional file 5: posterior distributions

Tables of posterior distributions

We present here the posterior distributions of all parameters, first separately for each combination of model and antibiotic.

Ciprofloxacin

Single-step model

Parameter	Interpretation	Posterior median (95% credible interval)	Unit
beta (HMW)	Transmission rate (HMW)	1.65 (0.60-5.10)	Per year
beta (MSM)	Transmission rate (MSM)	2.32 (1.01-6.34)	Per year
tau (HMW)	Treatment rate (HMW)	0.77 (0.37-2.60)	Per year
tau (MSM)	Treatment rate (MSM)	1.33 (0.73-4.22)	Per year
nu	Spontaneous recovery rate	0.72 (0.03-3.87)	Per year
mu	Mutation rate	0.00071 (0.00003-0.00349)	Probability
epsilon	Reduced treatment efficacy	0.40 (0.02-0.81)	Probability

Multi-step model

Not done because of the lack of detail on MIC after 2009 in the data

Cefixime

Single-step model

Parameter	Interpretation	Posterior median (95% credible interval)	Unit
beta (HMW)	Transmission rate (HMW)	1.46 (0.39-4.68)	Per year
beta (MSM)	Transmission rate (MSM)	1.93 (0.66-5.91)	Per year
tau (HMW)	Treatment rate (HMW)	0.61 (0.18-2.02)	Per year
tau (MSM)	Treatment rate (MSM)	0.99 (0.35-3.43)	Per year
nu	Spontaneous recovery rate	0.72 (0.03-3.72)	Per year
mu	Mutation rate	0.00097 (0.00008-0.00370)	Probability
epsilon	Reduced treatment efficacy	0.67 (0.08-0.98)	Probability

Multi-step model

Parameter	Interpretation	Posterior median (95% credible interval)	Unit
beta (HMW)	Transmission rate (HMW)	3.97 (1.93-9.02)	Per year
beta (MSM)	Transmission rate (MSM)	5.09 (2.46-11.53)	Per year
tau (HMW)	Treatment rate (HMW)	3.69 (1.72-8.75)	Per year
tau (MSM)	Treatment rate (MSM)	4.72 (2.21-11.05)	Per year
nu	Spontaneous recovery rate	0.21 (0.03-0.68)	Per year
mu	Mutation rate	0.04 (0.02-0.09)	Probability
epsilon	Reduced treatment efficacy	0.89 (0.65-0.98)	Probability

Azithromycin

Single-step model

Parameter	Interpretation	Posterior median (95% credible interval)	Unit
beta (HMW)	Transmission rate (HMW)	1.27 (0.30-4.54)	Per year
beta (MSM)	Transmission rate (MSM)	1.60 (0.42-5.15)	Per year
tau (HMW)	Treatment rate (HMW)	0.44 (0.13-1.61)	Per year
tau (MSM)	Treatment rate (MSM)	0.69 (0.20-2.60)	Per year
nu	Spontaneous recovery rate	0.65 (0.02-3.92)	Per year
mu	Mutation rate	0.00056 (0.00003-0.00255)	Probability
epsilon	Reduced treatment efficacy	0.87 (0.30-0.99)	Probability

Multi-step model

Parameter	Interpretation	Posterior median (95% credible interval)	Unit
beta (HMW)	Transmission rate (HMW)	1.97 (0.90-4.80)	Per year
beta (MSM)	Transmission rate (MSM)	2.19 (1.02-5.40)	Per year
tau (HMW)	Treatment rate (HMW)	1.70 (0.70-4.48)	Per year
tau (MSM)	Treatment rate (MSM)	1.88 (0.83-5.00)	Per year
nu	Spontaneous recovery rate	0.22 (0.03-0.71)	Per year
mu	Mutation rate	0.03 (0.01-0.06)	Probability
epsilon	Reduced treatment efficacy	0.98 (0.91-1.00)	Probability

Ceftriaxone

Single-step model

Parameter	Interpretation	Posterior median (95% credible interval)	Unit
beta (HMW)	Transmission rate (HMW)	1.08 (0.22-4.12)	Per year
beta (MSM)	Transmission rate (MSM)	1.64 (0.41-5.29)	Per year
tau (HMW)	Treatment rate (HMW)	0.29 (0.07-1.17)	Per year
tau (MSM)	Treatment rate (MSM)	0.68 (0.18-2.99)	Per year
nu	Spontaneous recovery rate	0.68 (0.03-3.59)	Per year
mu	Mutation rate	0.00022 (0.00000-0.00168)	Probability
epsilon	Reduced treatment efficacy	0.91 (0.31-1.00)	Probability

Multi-step model

Parameter	Interpretation	Posterior median (95% credible interval)	Unit
beta (HMW)	Transmission rate (HMW)	4.21 (1.32-9.67)	Per year
beta (MSM)	Transmission rate (MSM)	4.55 (1.41-10.42)	Per year
tau (HMW)	Treatment rate (HMW)	3.95 (1.08-9.38)	Per year
tau (MSM)	Treatment rate (MSM)	4.23 (1.13-9.92)	Per year
nu	Spontaneous recovery rate	0.21 (0.03-0.68)	Per year
mu	Mutation rate	0.04 (0.01-0.09)	Probability
epsilon	Reduced treatment efficacy	0.99 (0.36-1.00)	Probability

#'

Plots of posterior distributions

For each combination of model and antibiotic, we show the summary of the posterior distributions (median and 95% credible interval) of all parameters. We also present pairs plots showing the correlation between parameters. The observed relationships between parameters are in line with what is expected given the model structure: high correlation between β, τ and μ within a population as it is the combination of these parameters that determines resistance, and high correlation between β across groups because of the constraint that τ must be higher for MSM than for HMW.

Single-step models

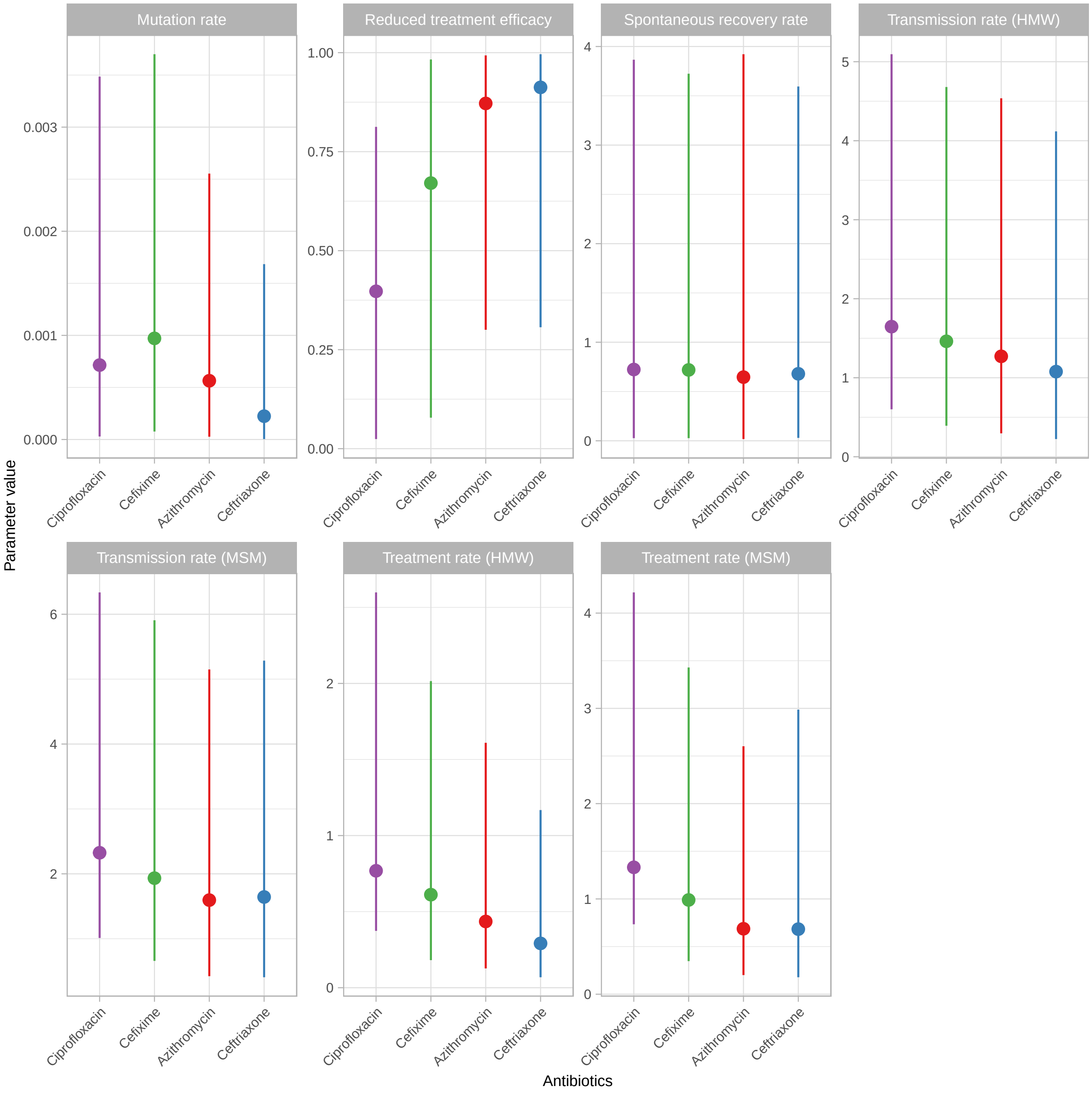

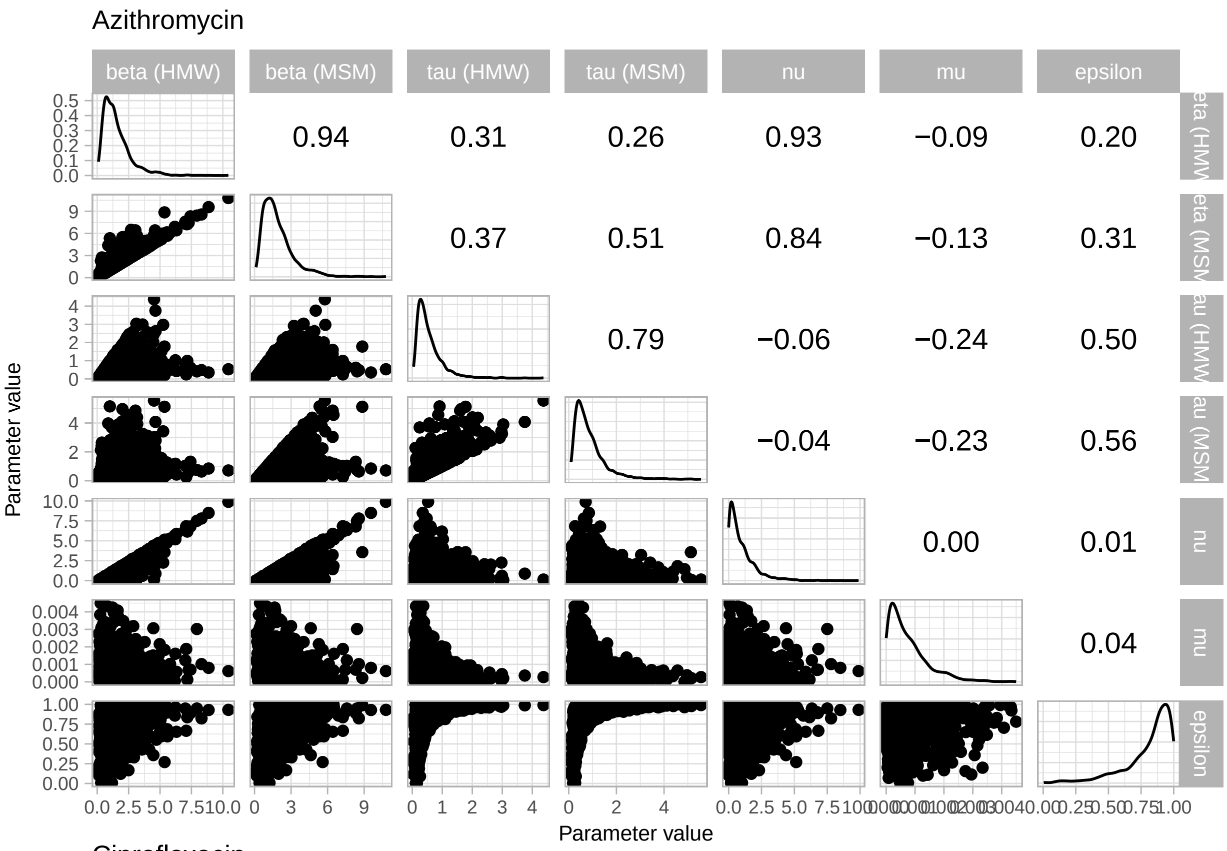
Azithromycin
beta (HMW)
beta (MSM)
tau (HMW)
tau (MSM)
nu
mu
epsilon
0.94
0.31
0.26
0.93
−0.09
0.20
0.37
0.51
0.84
−0.13
0.31
0.79
−0.06
−0.24
0.50
−0.04
−0.23
0.56
0.00
0.01
0.04
Parameter value
Parameter value

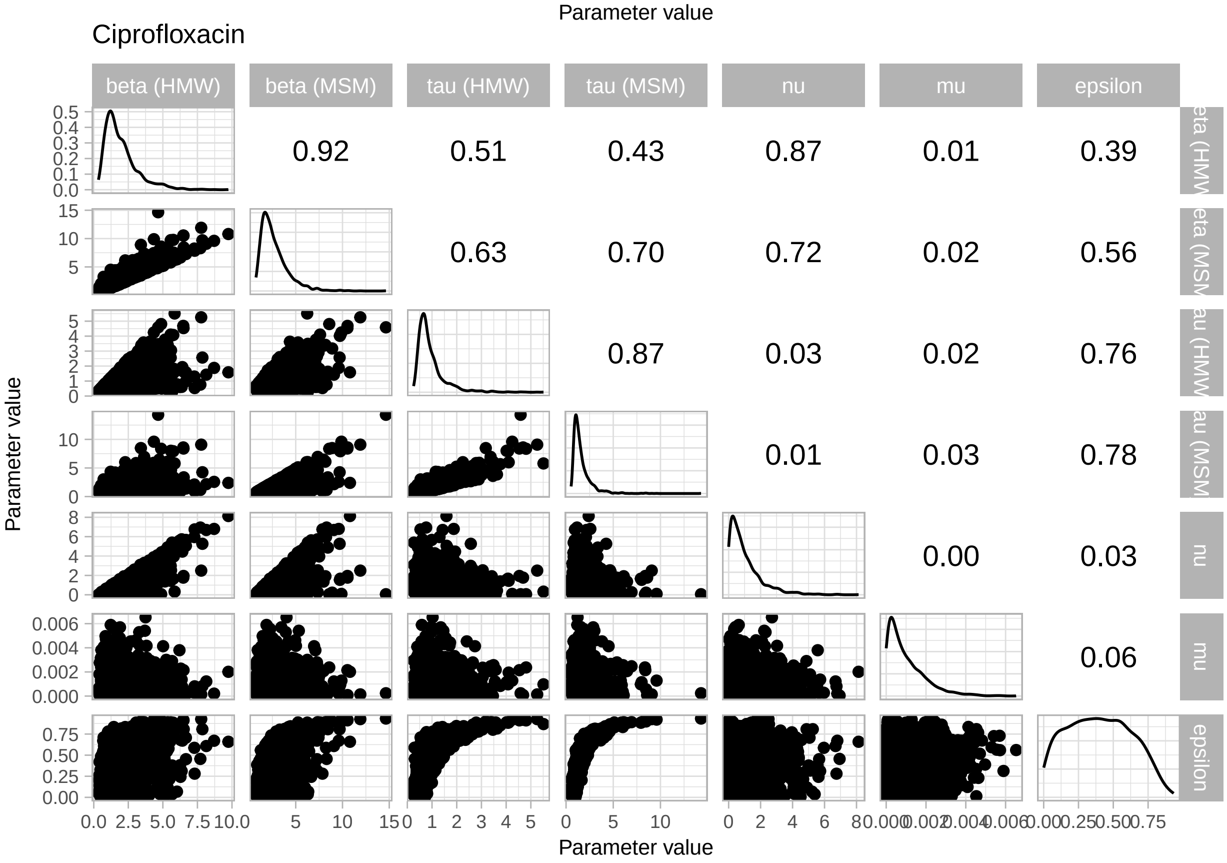
Ciprofloxacin
beta (HMW)
beta (MSM)
tau (HMW)
tau (MSM)
nu
mu
epsilon
0.92
0.51
0.43
0.87
0.01
0.39
0.63
0.70
0.72
0.02
0.56
0.87
0.03
0.02
0.76
0.01
0.03
0.78
0.00
0.03
0.06
Parameter value
Parameter value

Cefixime

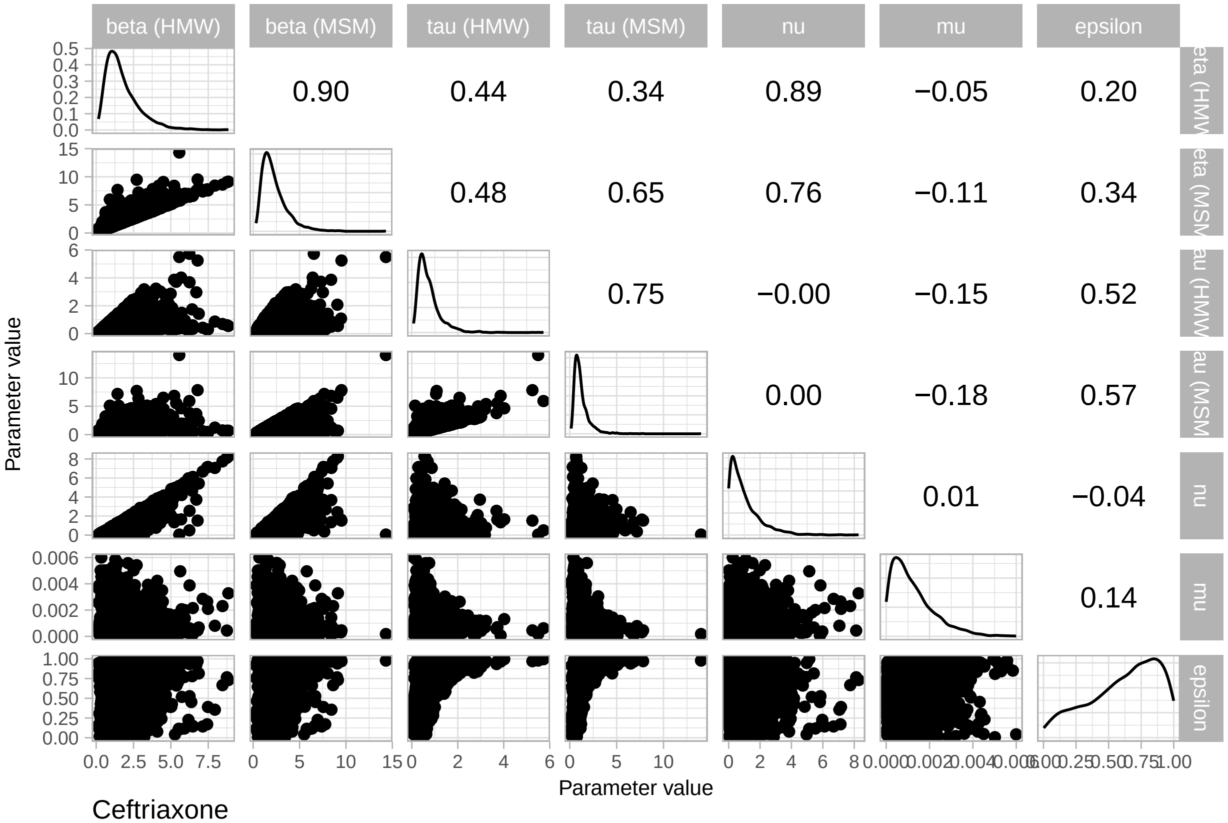

Ceftriaxone

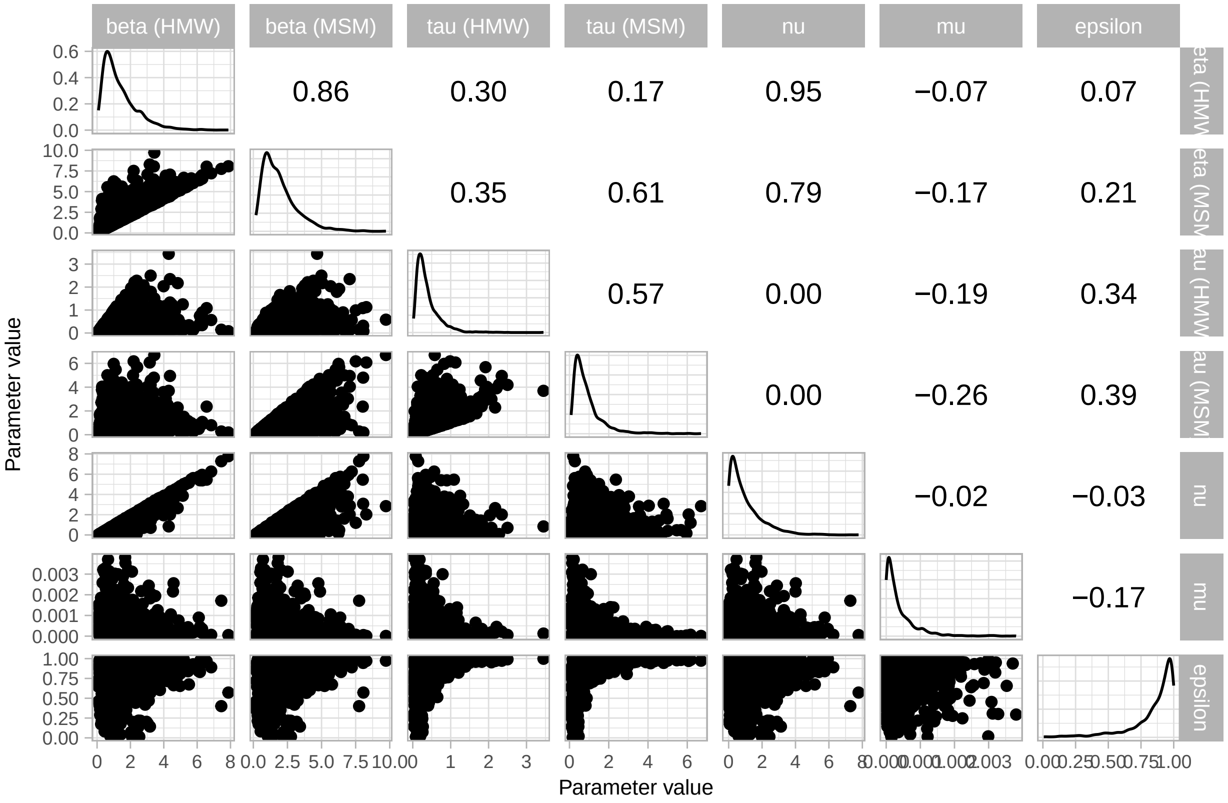

Multi-step models

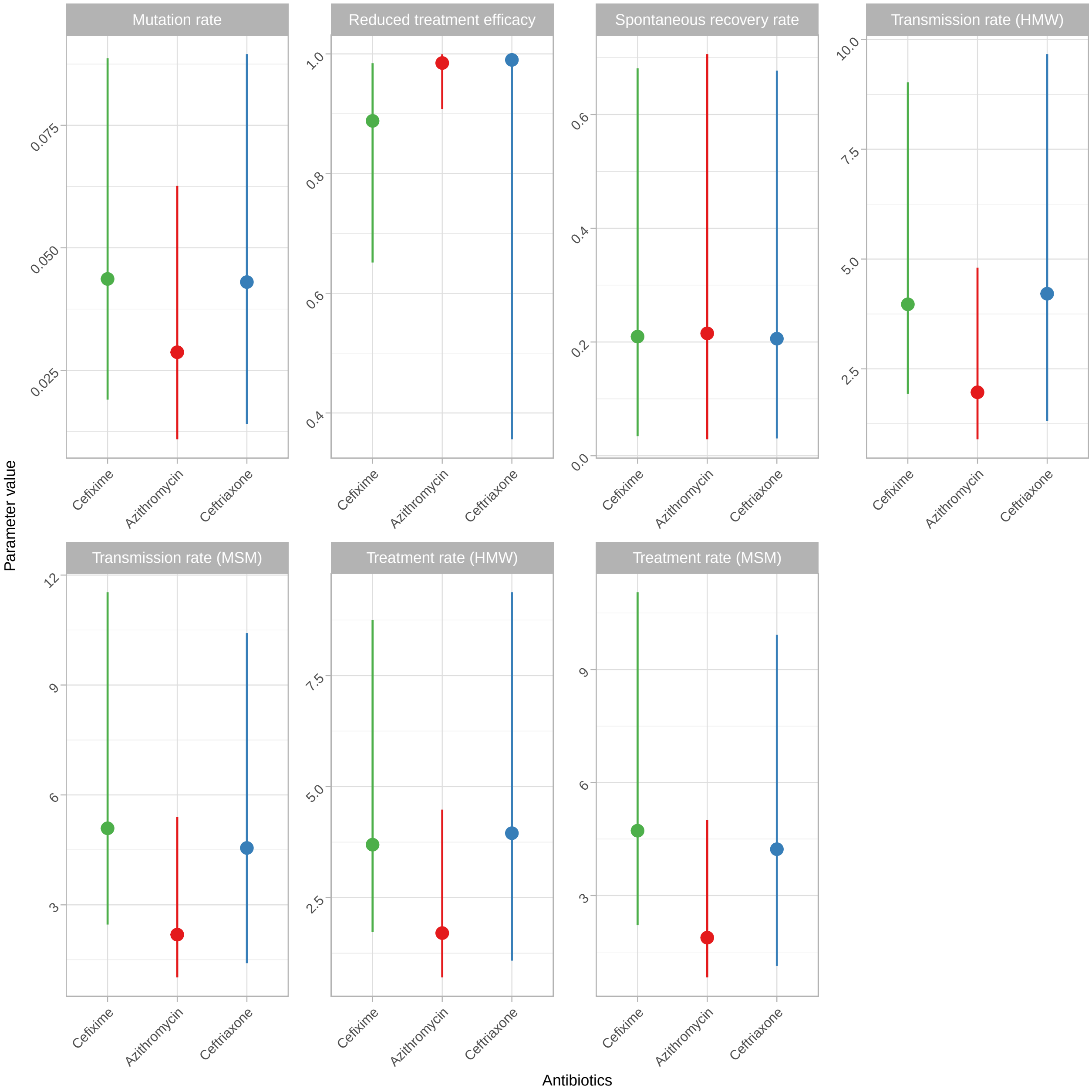

Azithromycin

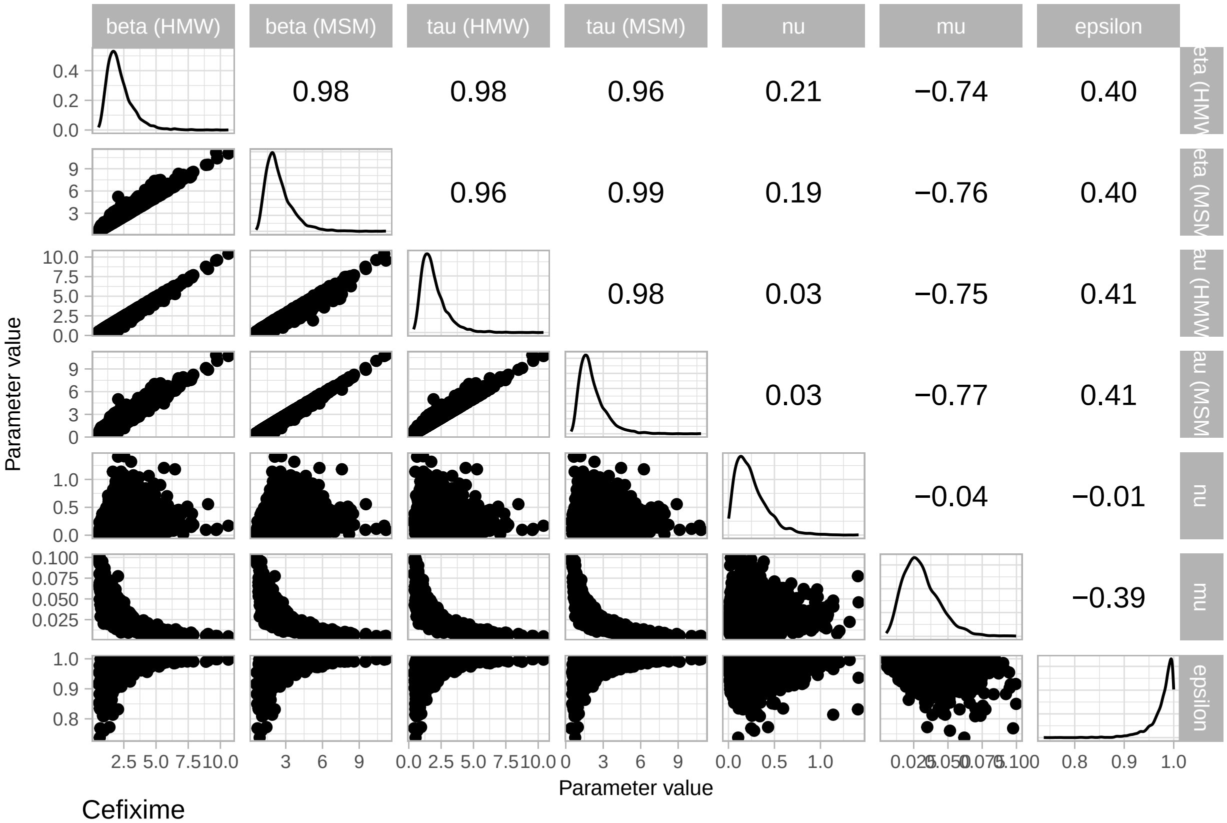

Cefixime

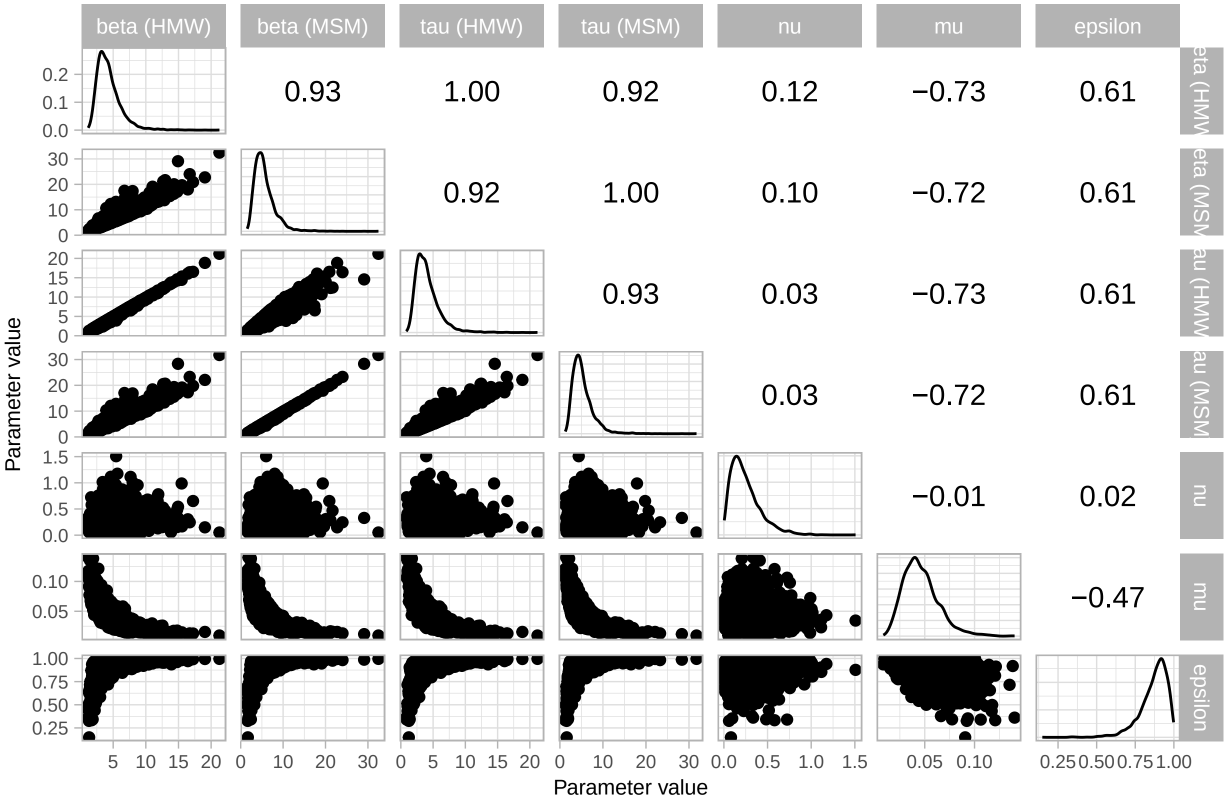

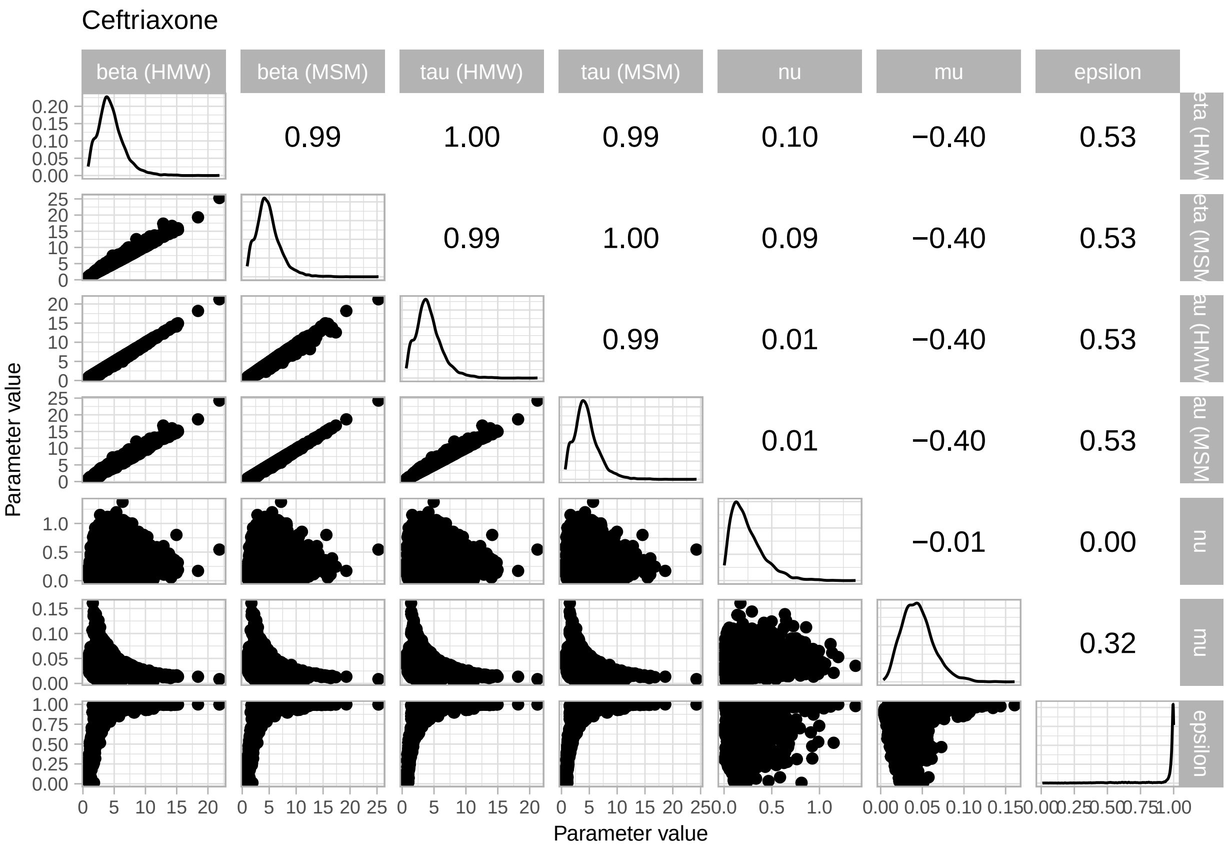
Ceftriaxone
beta (HMW)
beta (MSM)
tau (HMW)
tau (MSM)
nu
mu
epsilon
0.99
1.00
0.99
0.10
−0.40
0.53
0.99
1.00
0.09
−0.40
0.53
0.99
0.01
−0.40
0.53
0.01
−0.40
0.53
−0.01
0.00
0.32
Parameter value
Parameter value
